# Enhanced Metabolic Risks in Patients with Obesity and Nutritional Deficiencies in the U.S.: A TriNetX Study

**DOI:** 10.3390/nu18183005

**Published:** 2026-09-14

**Authors:** Elvia Y. Osorio, Zbigniew Gugala, Peter C. Melby, Monique R. Ferguson, Kamil Khanipov

**Affiliations:** 1 Department of Internal Medicine, Division of Infectious Diseases, University of Texas Medical Branch, Galveston, TX 77555, USA; pcmelby@utmb.edu (P.C.M.); mrfergus@utmb.edu (M.R.F.); 2 Department of Orthopedic Surgery and Rehabilitation, University of Texas Medical Branch, Galveston, TX 77555, USA; zgugala@utmb.edu; 3 Department of Microbiology and Immunology, University of Texas Medical Branch, Galveston, TX 77555, USA; 4 Center for Tropical Diseases, Institute for Human Infection and Immunity, University of Texas Medical Branch, Galveston, TX 77555, USA; 5 Department of Pharmacology and Toxicology, University of Texas Medical Branch, Galveston, TX 77555, USA; kakhanip@utmb.edu; 6 The Institute for Translational Sciences, University of Texas Medical Branch, Galveston, TX 77555, USA

**Keywords:** obesity, malnutrition, double burden of malnutrition, diabetes, metabolic syndrome, MASLD, MASH, electronic health records

## Abstract

Background/Objectives: Nutrient deficiencies associated with progression from obesity to metabolic dysfunction are poorly characterized. Using electronic health records (TriNetX Research Network), we evaluated the association between key nutritional deficiencies and metabolic outcomes among patients with obesity. Methods: Deficiencies assessed included protein–energy malnutrition, deficiencies in vitamin D, calcium, magnesium, and vitamin B12, and prior underweight status. Outcomes included severe obesity, prediabetes, type 2 diabetes, metabolic syndrome, metabolic dysfunction-associated steatotic liver disease (MASLD), and metabolic dysfunction-associated steatohepatitis (MASH). After matching propensity scores, cohorts with the double burden of malnutrition (DBM) were compared with a reference cohort of patients with obesity but no history of nutritional deficiencies. Results: The most prevalent types of DBM were vitamin D deficiency (10.6%), protein deficiency (8.9%) and calcium deficiency (7.5%). Incidence rates of obesity and all types of DBM increased from 2023 to 2025. Although middle-aged White and Black/African American patients represented the largest demographic groups, adults aged >70 years exhibited higher incidence rates of protein, calcium, magnesium, and B12 deficiencies. Vitamin D deficiency (median hazard ratio [HR] 3.09; IQR 2.32–5.66), vitamin B12 deficiency (median HR 2.49; IQR 2.16–6.08), and prior underweight (median HR 2.23; IQR 1.76–3.98) were associated with the highest burden of metabolic diseases. Conclusions: These findings underscore the interplay between nutritional deficiencies and metabolic disease risk in obesity and support targeted interventions for populations affected by DBM, especially older adults, and in the context of racial and socioeconomic disparities.

## 1. Introduction

The double burden of malnutrition (DBM), which is the coexistence of undernutrition and obesity or overweight within the same population, household, or individual, is a public health challenge in low- and middle-income countries [1,2,3]. However, its impact in high-income and developed countries is less established. From 1990 to 2022, the prevalence of obesity increased more rapidly globally than the prevalence of underweight, making obesity the dominant driver of the DBM worldwide [2,4]. Although there is no single estimate of the global incidence and prevalence of the DBM [5], it can be inferred from the prevalence of undernutrition and overweight. A meta-analysis from the NCD Risk Factor Collaboration estimated that in 2022, across 200 countries, 10–15% of adults in South Asia and Sub-Saharan Africa were underweight, whereas obesity rates were 7–25% in most low- and middle-income countries. In contrast, in high-income countries, less than 5% were underweight, but obesity exceeded 20–25% in children/adolescents and 30% in adults [4]. National Health and Nutrition Examination Survey (NHANES) data indicate that the prevalence of obesity among U.S. adults increased from 30.5% in 1999–2000 to 40.3% during 2021–2023. CDC state-level surveillance data from 2024, showed that obesity prevalence ranged from 30% to <35% in 22 states, 35% to <40% in 17 states, and 25% to <30% in 8 states, with the highest burden concentrated in the Southern and Midwestern regions [6,7]. NHANES data from 2002 to 2008 demonstrated that adults with obesity had a 20% or higher prevalence of micronutrient inadequacies, for vitamin C, vitamin D, vitamin E, calcium and magnesium than normal-weight adults [8]. Studies in bariatric surgery candidates have highlighted the high burden of malnutrition within this population, reporting that up to 85.5% of patients presented at least one nutritional deficiency before surgery, and that vitamin D deficiency affected 70–90% of patients [9]. However, establishing consensus estimates for the national prevalence of the DBM remains challenging, largely because its identification relies on diverse combinations of anthropometric, biochemical, and clinical indicators.

Nutritional deficiencies likely increase susceptibility to metabolic diseases [10]. Exposure to prenatal or childhood famine increases the risk of adult hyperglycemia and type 2 diabetes [11,12,13,14]; therefore, the metabolic imprint of early nutritional deprivation persists into adulthood and shapes lifelong susceptibility to metabolic dysfunction. A second major form of DBM beyond the underweight–overweight pattern is the “hidden hunger” of malnutrition, driven by micronutrient deficiencies [15]. In general, the DBM arises from food insecurity, socioeconomic disparities [16], dietary transitions associated with urbanization [17], and dietary habits associated with the adoption of hyperpalatable, ultra-processed, calorie-dense diets [3,18]. Ultra-processed foods, high in refined sugars, saturated fats, and sodium but low in essential micronutrients and fiber, promote excess caloric consumption and weight gain [19] while simultaneously contributing to inadequate micronutrient intake required for metabolic homeostasis [18]. Besides nutrients, lifestyle and environmental factors impair micronutrient metabolism and absorption [20], including reduced sunlight exposure [21], changes in the gut microbiome [22], circadian rhythm dysregulation [23], psychological and environmental stress [24,25]. Thus, micronutrient deficiency arises not only from diet but also from the broader metabolic constraints imposed by the obesogenic environment.

Micronutrient deficiencies are inherent in the pathophysiology of obesity [20,26,27]. Deficits in vitamin D, iron, zinc, and magnesium exacerbate insulin resistance, dyslipidemia, endothelial dysfunction, and chronic inflammation, thus amplifying metabolic risk [28,29]. Nevertheless, evolutionary theories suggest that adiposity may confer adaptive advantages, such as enhanced immune buffering, thermoregulation, and reproductive fitness, which vary by age, sex, and reproductive status, complicating the interpretation of obesity-related health effects across populations [30]. Further, the extent of nutritional deficiencies in obesity and their contribution to non-communicable metabolic disease is poorly characterized. To address this gap, we evaluated the association of key obesity-related nutritional deficiencies with major metabolic comorbidities in predominantly U.S. cohorts and estimated their contribution to the risk of major metabolic diseases. The TriNetX research network used in this study contains electronic health records from millions of patients across U.S. healthcare organizations, including longitudinal clinical data spanning up to approximately two decades for many individuals, facilitating large-scale evaluation of obesity-associated nutritional deficiencies in healthcare settings and assess their long-term associations with metabolic disease burden.

## 2. Materials and Methods

Cohorts: We used publicly available data from December 2025 to February 2026 from the TriNetX platform, a research network which provides access to longitudinal electronic health records from participating healthcare organizations, with records for some patients spanning back approximately 20 years. This secondary analysis of TriNetX data does not involve interaction with human subjects. Any personally identifiable information was de-identified as defined in Section 164.512(a) of the HIPAA Privacy Rule.

Inclusion criteria: Nutritional deficiencies were identified at the first obesity diagnosis. The exception was the underweight cohort, which included patients diagnosed with underweight at least six months before the obesity index event, because underweight and obesity cannot coexist (Table 1). The inclusion criteria for the obesity cohort included the first documented diagnosis of childhood or adult obesity (Table S1). The age range was not restricted to a specific group. Nutritional deficiencies were defined by the presence of a documented diagnosis of the nutritional deficiency (Table 1) and/or low serum levels of protein or micronutrient deficiencies commonly associated with obesity, such as vitamin D, calcium, magnesium, and vitamin B12 (Table 1).

Exclusion criteria: Patients with confounding conditions associated with malnutrition were excluded, including those diagnosed with neoplasms, syndromic genetic obesity, ongoing pregnancy, and genetic and congenital disorders, among others (Table S2). Patients with confounding diseases or inflammatory conditions that could affect serum micronutrient levels were excluded (Table S3). Cohorts with iron, zinc, vitamin A, and folate deficiencies represented less than 0.1% of the population diagnosed with obesity and were excluded from further analysis.

Outcome analysis included a documented diagnosis of severe obesity (class 2 or class 3), prediabetes, and diabetes mellitus (Table S4). Metabolic dysfunction-associated steatohepatitis (MASH) was identified by the documented diagnosis of nonalcoholic steatohepatitis (NASH). Metabolic dysfunction-associated steatotic liver disease (MASLD) was identified as a documented diagnosis of fatty liver disease, both excluding alcoholic liver disease. Metabolic syndrome (MetS) was defined by a documented MetS diagnosis. The presence of at least two MetS components concomitant with obesity was evaluated as surrogate indicator of metabolic syndrome (Table S4).

**Figure 1:**
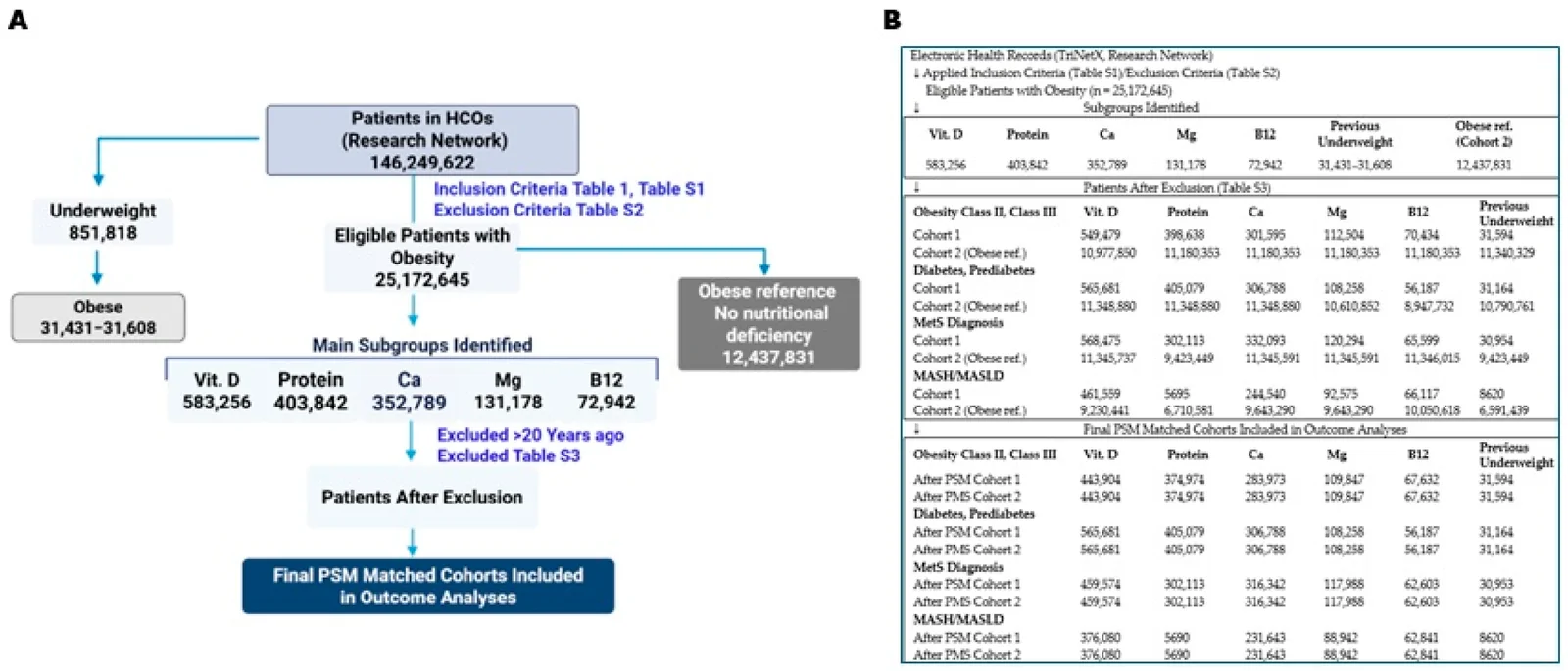
Cohort derivation of patients with obesity according to the most prevalent forms of the double burden of malnutrition for metabolic outcome analysis using electronic health records (TriNetX Platform, Research Network). (**A**) Schematic overview of cohort derivation and study design (created in BioRender. Osorio, E. (2026) https://BioRender.com/aphwzxg (accessed on 8 September 2026). (**B**) Patient counts after exclusion criteria and propensity score matching for outcome analysis. Patients with documented nutritional deficiencies identified on the same date as first obesity diagnosis (cohort 1) were compared with a reference cohort (cohort 2) of obese patients without a documented nutritional deficiency (Obese ref.).

Statistics: Outcomes were compared with a reference cohort of patients with obesity who had no history of nutritional deficiencies (obese reference cohort) (Figure 1). Initially, cohorts were balanced using propensity score matching (PSM) (1:1) for age, sex, race, obesity status/severity as well for use of medications within the hormones/synthetics/modifiers therapeutic class (HS000), including blood glucose-regulating agents, insulin, metformin, and other endocrine therapies. Use of the cardiovascular medications class (CV000), antihypertensive, antiarrhythmic, lipid-lowering drugs and other cardiovascular agents was also included in the PSM. Because nutritional deficiencies have long-term effects, and evidence suggests that the metabolic consequences of malnutrition may persist for decades after the episode [11,31,32,33], we analyzed outcomes from one day to any time after the first index event of obesity. Patients with outcomes >20 years prior to the index event, first diagnosis of obesity, were excluded from the outcome analysis. Propensity score matching, Kaplan–Meier survival curves, odds ratios (ORs), risks ratios (RRs), risk difference (RD), incidence, and prevalence were calculated automatically by the TriNetX analytics platform. Sensitivity analyses were done considering PSM for body mass index (BMI) instead of obesity status/severity and BMI plus the presence of the laboratory value of interest. Results were compared with the initial analysis to assess the robustness of the observed associations.

Incidence or prevalence stratified by race was calculated as the number of incident or prevalent cases within each racial group divided by the population at risk in that racial group. Z-scores for incidence rates were calculated by subtracting the mean incidence of the reference group within each age group and dividing the result by the standard deviation of the reference group (z = [X − μ_ref]/σ_ref). Standardized residuals were calculated as the difference between the observed and expected counts divided by the square root of the expected count [34].

## 3. Results

### 3.1. Trends in Incidence and Prevalence of Nutrient Deficiencies

**Figure 2:**
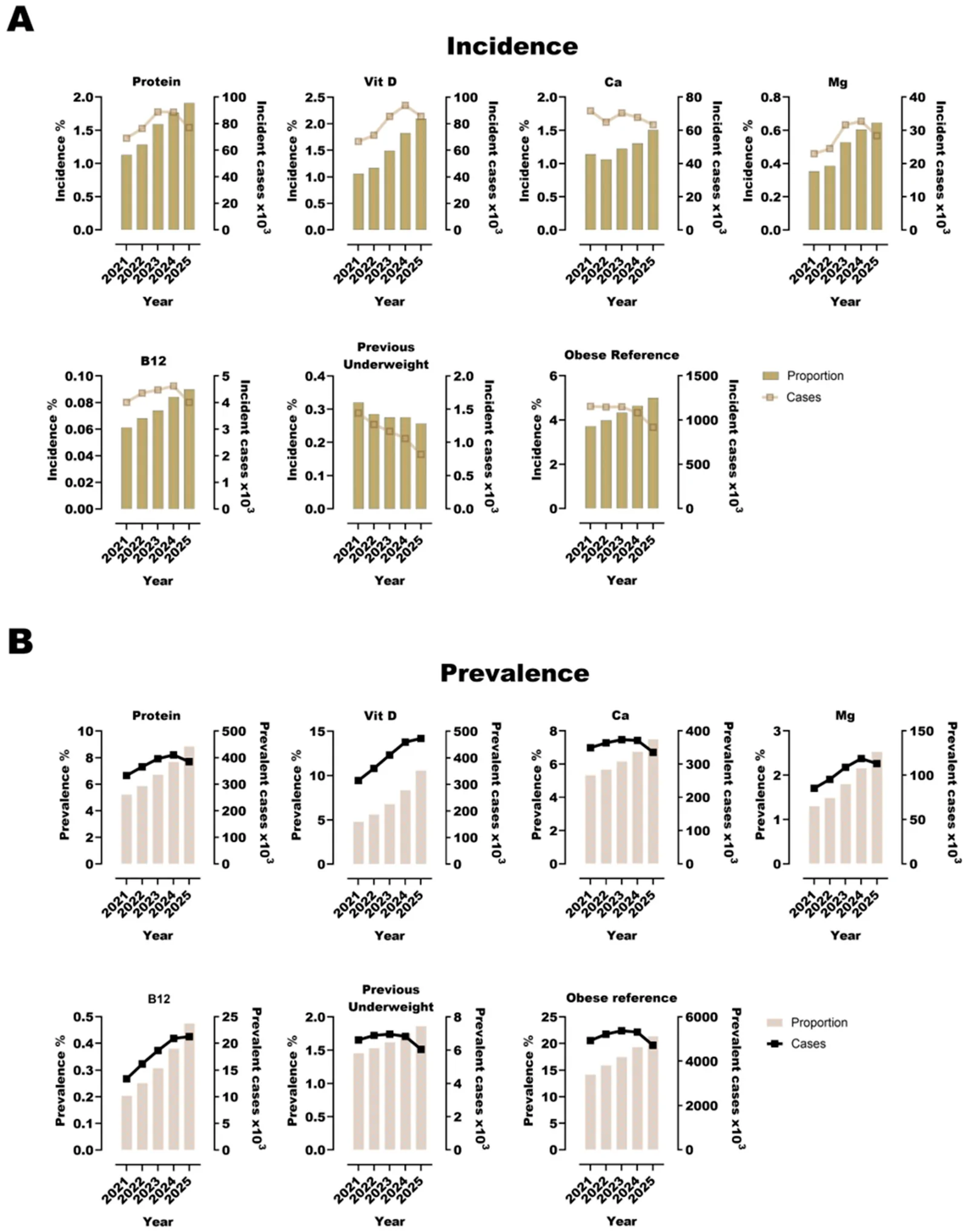
Incidence and prevalence of primary nutritional deficiencies among patients with obesity based on electronic health records from the TriNetX Research Network, 2021 to 2025. (**A**) Incidence from 2021 to 2025. (**B**) Prevalence from 2021 to 2025. Each deficiency in patients diagnosed with obesity and nutritional deficiencies, or in the reference group without nutritional deficiencies. Bars: percentage ((**left**), *y*-axis); line: number of cases ((**right**), *y*-axis).

**Figure 3:**
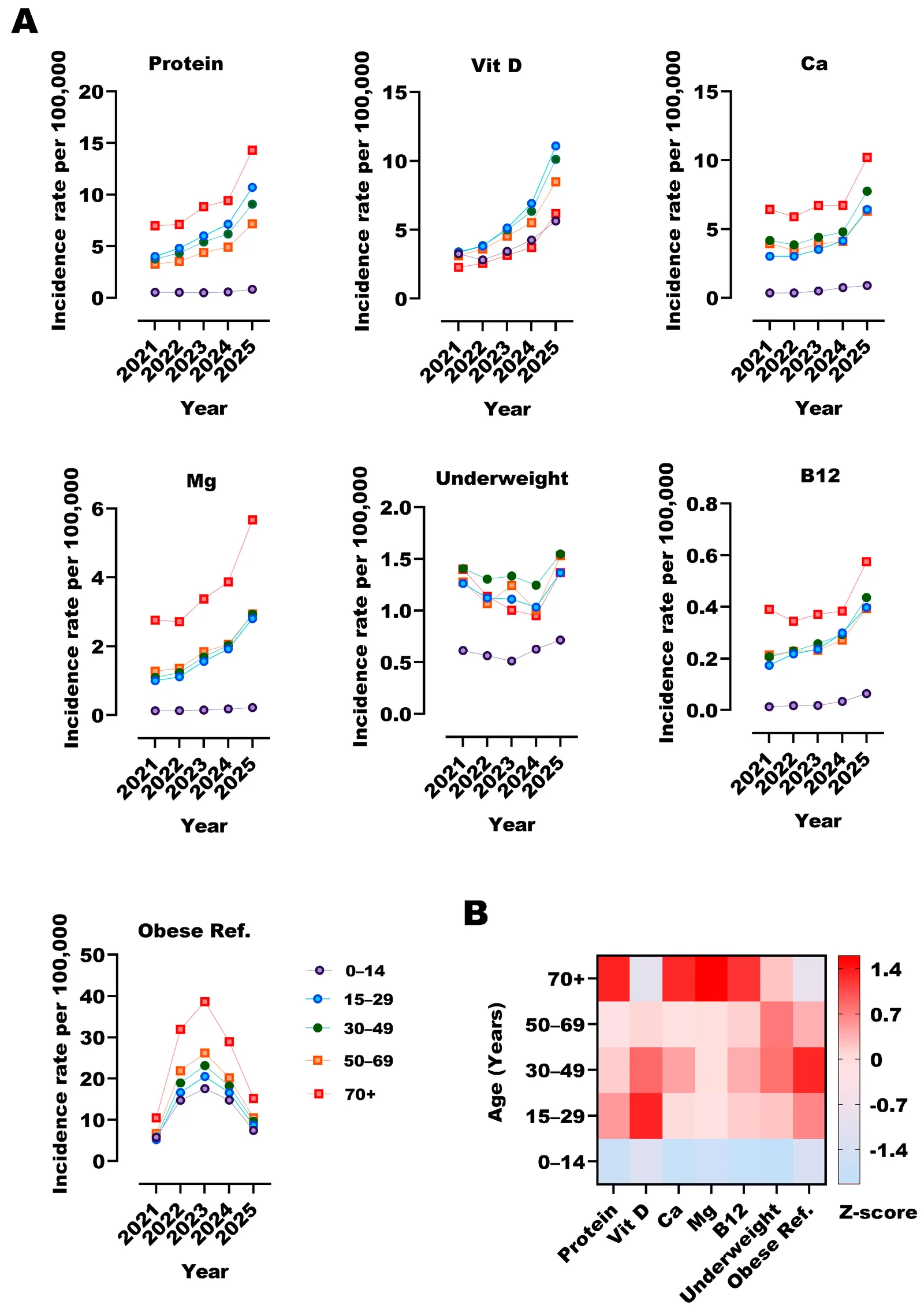
Incidence rates of nutritional deficiencies by age group in patients with obesity based on electronic health records from the TriNetX Research Network, from 2021 to 2025, showing higher rates in patients >70 years. (**A**) Incidence rate of nutritional deficiencies per 100,000 patients diagnosed from 2021 to 2025. (**B**) Heat map incidence patterns by nutrient deficiency for the indicated age ranges. Incidence rates are shown as Z-scores standardized to a reference group without nutritional deficiencies (obese ref.). Positive values indicate higher incidence, and negative values indicate lower incidence relative to the incidence of the cohort without nutritional deficiencies.

Protein, vitamin D, calcium, magnesium, and vitamin B12 deficiencies, as well as a history of prior underweight, were the most prevalent in patients diagnosed with obesity (Figure 1). Vitamin D deficiency was the most prevalent (10.6%) (Figure 2A,B), followed by protein deficiency (8.9%) (Table 2). The incidence rates of newly diagnosed obesity with or without nutritional deficiencies increased from 2023 to 2025 (Figure 3A, Table S5). Despite absolute case counts declined in 2025 (Figure 2), this pattern was observed across multiple cohorts and likely reflects changes in the underlying population represented in the database rather than a true decline in disease occurrence.

The incidence rates of protein, calcium, magnesium, and vitamin B12 deficiencies were highest among adults aged ≥70 years. Previous underweight, vitamin D deficiency, and the reference obese cohort without nutritional deficiencies were highest in adolescents and younger adults (Figure 3B).

### 3.2. Demographics

Data were assessed to identify at-risk populations and disparities across groups.

#### 3.2.1. Age Distribution

**Figure 4:**
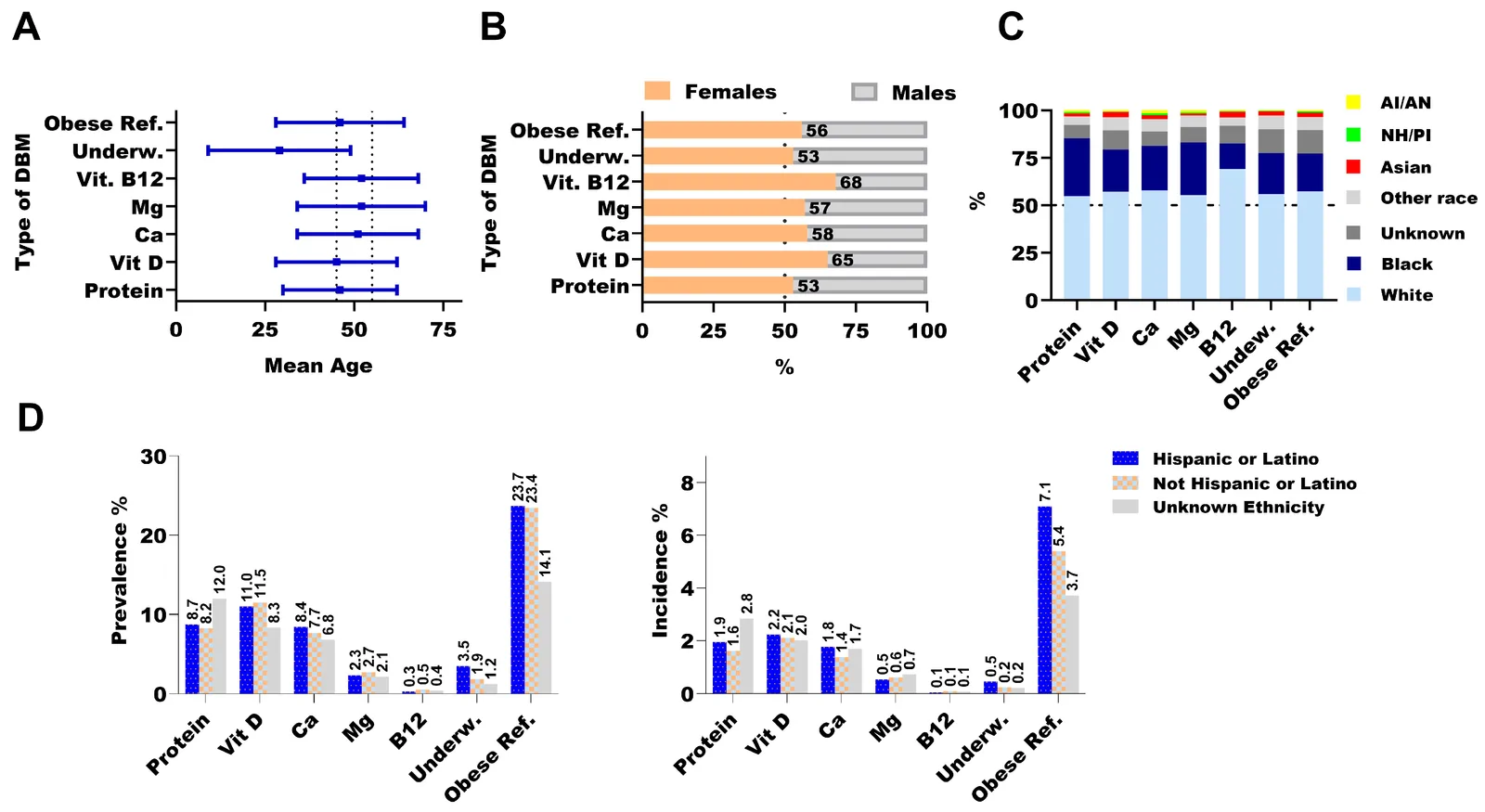
Nutritional deficiencies by age, sex, race, and ethnicity show a higher frequency in Black and AI/AN patients. (**A**) Mean age (±standard deviation) of individuals with nutritional deficiencies or the reference group without nutritional deficiencies (obese ref.). Vertical lines indicate ages 45 and 55. (**B**) Sex distribution of individuals with nutritional deficiencies or the reference group showing overrepresentation in females. Dots on bars show 50%. (**C**) Distribution by race of patients with obesity and nutritional deficiencies or the reference group. AI/AN, American Indian or Alaska Native; NH/PI, Native Hawaiian or Other Pacific Islander. (**D**) Prevalence and incidence rates stratified by ethnicity.

The youngest cohort of patients at first diagnosis of obesity was the cohort with a history of underweight, with a mean age of about 29–39 years (Figure 4A). Protein and vitamin D deficiencies, and the obese reference cohort without nutritional deficiencies, clustered at a middle age of 45–48 years. Vitamin B12, calcium, and magnesium deficiencies also clustered in middle age, at 51–54 years (Figure 4A). Sex distribution: Female patients diagnosed with obesity were more highly represented across all categories of nutritional deficiencies (Figure 4B); however, protein deficiency showed the largest difference for females compared with obese males (Figure 4B).

#### 3.2.2. Ethnicity and Race

**Figure 5:**
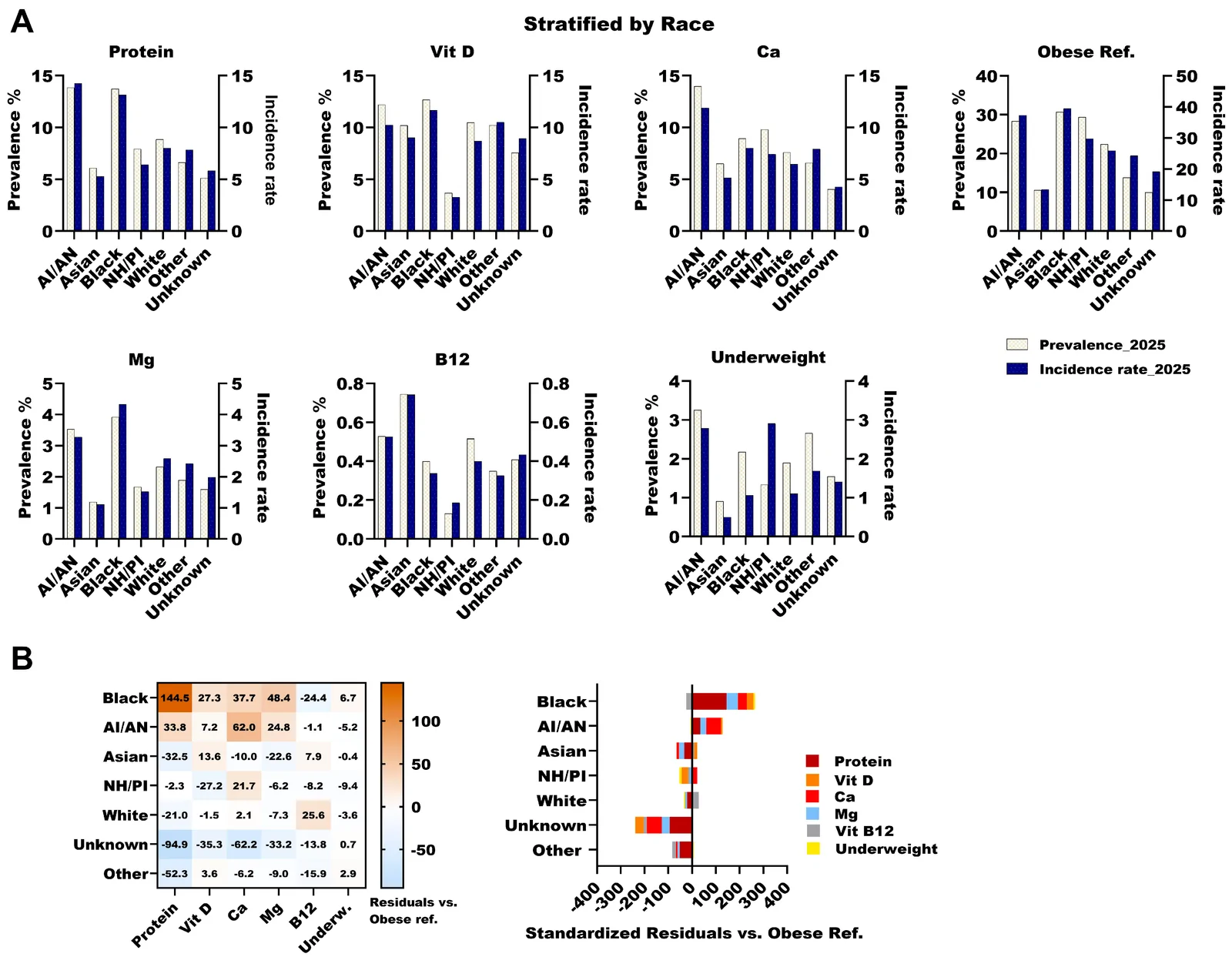
Adjusted estimates of nutritional deficiencies by race, 2025. (**A**) Prevalence and incidence rate stratified by race. (**B**) Standardized residuals to adjust for racial distribution relative to the reference group. Positive residuals (dark brown) indicate over-representation and negative residuals (light blue) indicate under-representation compared with the reference group. Absolute standardized residuals greater than 2.0 are considered significant. (**Left**): heat map, (**Right**): standardized residuals displayed as stacked bars, with each bar representing the sum of standardized residuals for each race. AI/AN, American Indian or Alaska Native; NH/PI, Native Hawaiian or Other Pacific Islander.

Patients of the white race comprised the highest proportion of patients diagnosed with obesity in every type of nutritional deficiency, followed by Black/African Americans (Figure 4C). To identify disparities, incidence and prevalence were estimated by the number of patients of each race. Black/African American and American Indian/Alaska Native patients showed disproportionately higher incidence and prevalence estimates across all groups, including the reference obese cohort without nutritional deficiencies (Figure 5A). In contrast, vitamin B12 deficiency was frequent in Asian patients with obesity. To uncover groups at risk of nutritional deficiencies, we calculated a standardized residual analysis against the expected proportions in the reference cohort of patients with obesity without history of nutritional deficiency. Protein, vitamin D, calcium, and magnesium deficiencies were over-represented in Black or African American patients and American Indian or Alaskan Natives. In contrast, vitamin B12 deficiency was over-represented in White patients (Figure 5B). Calcium deficiency was over-represented among Native Hawaiian/Pacific Islanders (Figure 5B). Stratification of incidence and prevalence by ethnicity showed a higher incidence of previous underweight, calcium deficiency, and obesity without nutritional deficiencies among Hispanic patients compared to non-Hispanics (Figure 4D).

#### 3.2.3. Geographical Distribution

**Figure 6:**
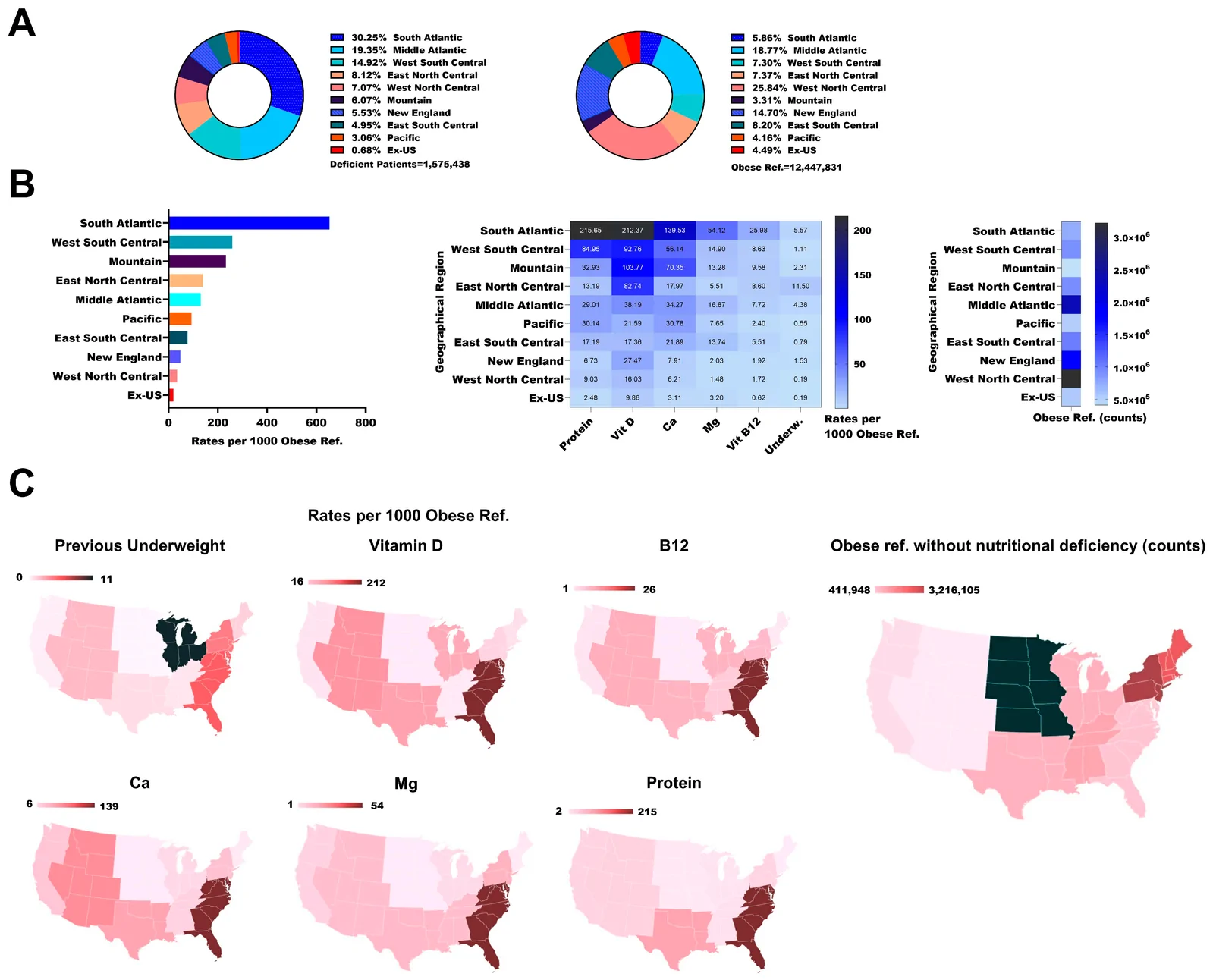
Geographical distribution of nutritional deficiencies in patients with obesity in the U.S., with the highest burden in the South Atlantic. (**A**) The U.S. geographical region distribution of patients with obesity and nutritional deficiencies (**left**) vs. the reference group (**right**). (**B**) Bar chart and heat map showing regional rates of nutritional deficiencies in patients with obesity (**left**) vs. total counts of those in the reference group per geographical region (**right**). Rates per 1000 obese = cases/cases in obese ref. cohort without nutritional deficiencies × 1000. (**C**) Choropleth maps showing the geographical distribution of patients with nutritional deficiencies colored as regional rates (cases per 1000 obese references). The obese reference cohort without nutritional deficiencies is colored as the number of patients (counts). Data from healthcare organizations located in the U.S., with exception of Ex-US, located outside the U.S. Choropleth maps with Datawrapper GmbH, Berlin, Germany.

The distribution of patients with obesity and associated nutritional deficiencies varied across U.S. geographic regions (Figure 6A). The highest cumulative burden of obesity with nutritional deficiencies occurred in the South Atlantic, Middle Atlantic, and West South Central regions (Table 3, Figure 6A). To reduce bias due to regional population size, the data were normalized per 1000 patients in the reference cohort. The South Atlantic and West South Central consistently showed the highest rates of nutritional deficiencies, followed by the Mountain regions (Figure 6B). The highest rates of patients with obesity and previous underweight were in the East North Central region, accompanied by high rates of protein and vitamin B12 deficiencies (Figure 6C).

### 3.3. Laboratory Data

**Figure 7:**
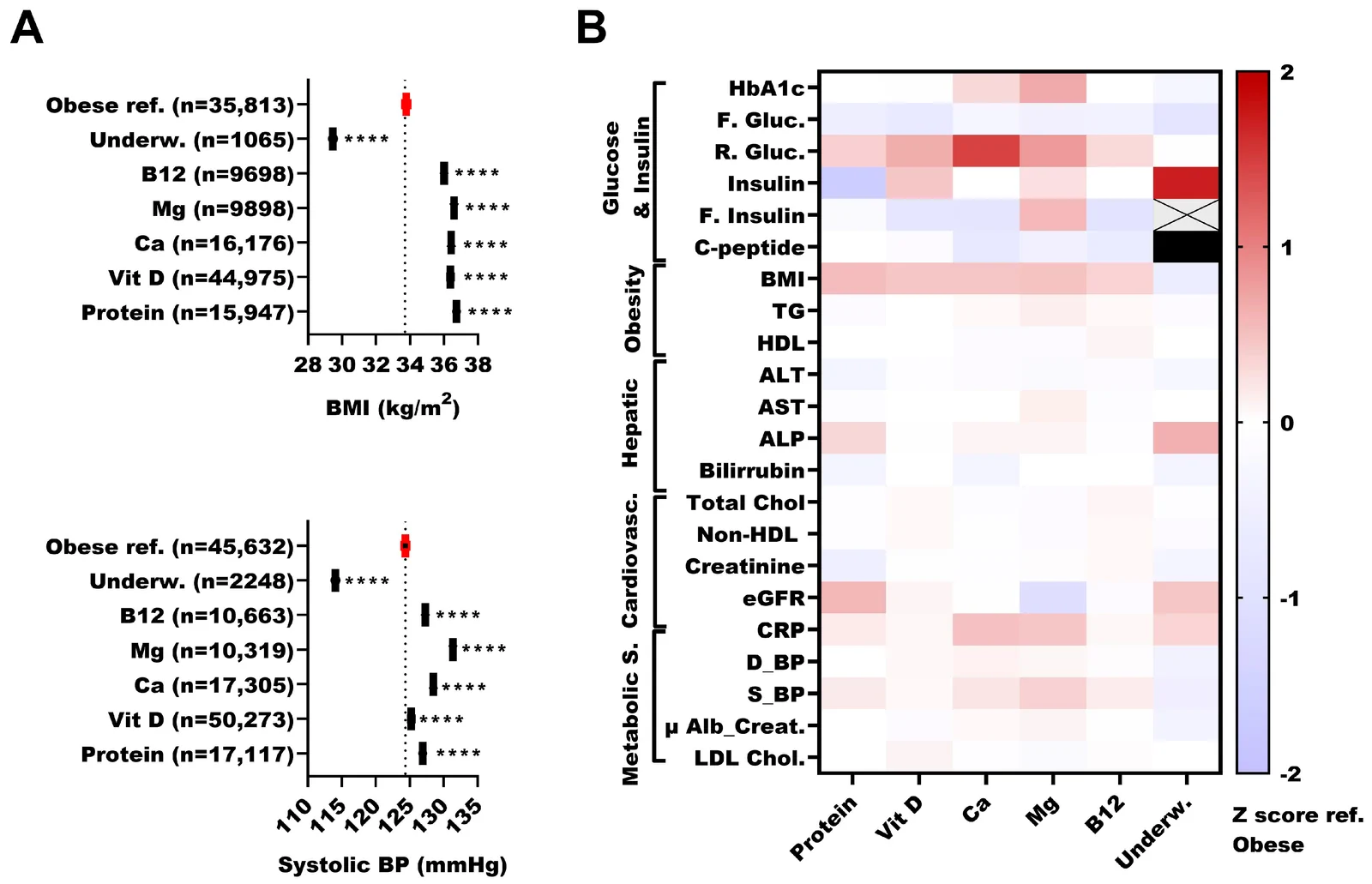
At first diagnosis of obesity, patients with a history of nutritional deficiencies exhibited higher BMI and systolic blood pressure compared with patients without history of nutritional deficiencies. (**A**) Body mass index (BMI) and systolic blood pressure (systolic BP). Data are shown as the mean (±standard deviation), n = number of patients, all comparisons vs. the reference group without history of nutritional deficiency (obese ref.), **** *p* < 0.0001. One-way Anova, Dunnett’s multiple comparison test. (**B**) Heat map showing a Z-score of laboratory values calculated from the difference between the median of each laboratory value in the nutritional deficiency group and the median of the reference group, standardized by the interquartile ranges (IQRs) in the reference group (IQR/1.349). Positive Z-scores indicate higher median values compared with the reference group, whereas negative Z-scores indicate lower median values. |Z| ≈ 0.2, small; 0.5, moderate; ≥0.8, large difference relative to the reference group. Hemoglobin A1c (HbA1c), fasting glucose (F. Gluc), random glucose (R. Gluc), fasting insulin (F. insulin), body mass index (BMI), triglycerides (TG), high-density lipoprotein (HDL), alanine aminotransferase (ALT), aspartate aminotransferase (AST), alkaline phosphatase (ALP), total cholesterol (T. Chol), non-HDL cholesterol (non-HDL), glomerular filtration rate (eGFR), *C*-reactive protein (CRP), systolic blood pressure (S_BP), diastolic blood pressure (D_BP), microalbumin/creatinine in urine (µ Alb_Creat.), low-density lipoprotein (LDL Chol).

To determine whether laboratory markers associated with metabolic disease differ at the time of the first diagnosis of obesity, we analyzed results collected within three months of diagnosis (Table S6). All groups of patients with obesity and nutritional deficiencies had a slightly but significantly higher body mass index (BMI) and systolic blood pressure than the reference cohort without a history of nutritional deficiencies (Figure 7A). Although most differences were small, they were statistically significant, likely due to the large sample size of each group (Figure 7A and Figure S1). To compare nutritional deficiencies, laboratory values were standardized to the obese cohort without nutritional deficiencies and expressed as Z-scores. Z-score analysis showed increased glycemia and lower insulin and *C*-peptide in magnesium, calcium, and vitamin B12 deficiencies compared with the obese reference group without nutritional deficiencies, indicating impaired pancreatic β-cell secretory function (Figure 7B). Patients with calcium, magnesium, and protein deficiency showed enhanced levels of the inflammatory marker CRP and higher systolic blood pressure than those in the reference cohort, indicative of hypertensive metabolic risk associated with inflammation (Figure 7B and Figure S1). In contrast, the previous underweight group showed a bias toward lower glycemic indices and higher insulin and *C*-peptide levels, suggestive of a prediabetic state (Figure 7B and Figure S1). However, because the previous underweight group had a small sample size, additional studies with larger cohorts are needed to confirm this observation.

### 3.4. Sensitivity Analysis

The initial propensity score matching (PSM) model balanced cohorts using obesity/severity rather than BMI to minimize potential overadjustment. To further reduce bias related to obesity duration and severity, the first recorded obesity diagnosis was used as the index event. However, because BMI was modest but significantly higher among individuals with nutritional deficiencies (Figure 7), we performed additional sensitivity analyses using PSM based on BMI. We found that matching on BMI strengthened the observed associations compared with the initial model (Tables S8 and S9). This suggests that the observed associations are unlikely to be explained solely by differences in adiposity burden. Hazard ratios generally followed a similar pattern, with most estimates being larger after matching directly on BMI than after matching on obesity status/severity (Table S9). More, few outcomes exhibited slight discrepancies between cumulative incidence measures RR/OR and HR, including obesity class 2 with protein deficiency and prediabetes with magnesium deficiency in the initial PSM model (Tables S8 and S9). While such differences may reflect the distinct aspects captured by cumulative incidence and time-to-event analyses, their resolution after BMI-based propensity score matching suggests that BMI was a more appropriate matching variable for these associations.

The exception is the group with a history of underweight status which consistently showed attenuation of effect estimates after BMI-based matching, and some of them lost statistical significance (Tables S8 and S9). Because these individuals begin follow-up from a substantially lower baseline body weight, matching directly on BMI may represent overadjustment.

Sensitivity analyses incorporated the presence of the nutrient specific laboratory test to minimize bias arising from differential screening and diagnosis practices. The results from this BMI and laboratory-adjusted PSM model produced results largely comparable to those obtained with BMI matching alone (Tables S8 and S9). Overall, these findings support the robustness of the observed associations across alternative approaches, while indicating that the magnitude of many associations is amplified when BMI is used as the matching variable with exception of prior underweight status. Therefore, the BMI and laboratory-adjusted PSM model was utilized for outcome analysis, except for prior underweight status, analyzed with the initial obesity severity-matched PSM model.

### 3.5. Outcomes

To assess the relevance of early laboratory predictors at the time of first obesity diagnosis and to assess subsequent metabolic disease risk, we calculated odds ratios, RRs and risk difference (RD) for each cohort with nutritional deficiencies, using the obese cohort with no history of deficiencies as the reference group.

**Figure 8:**
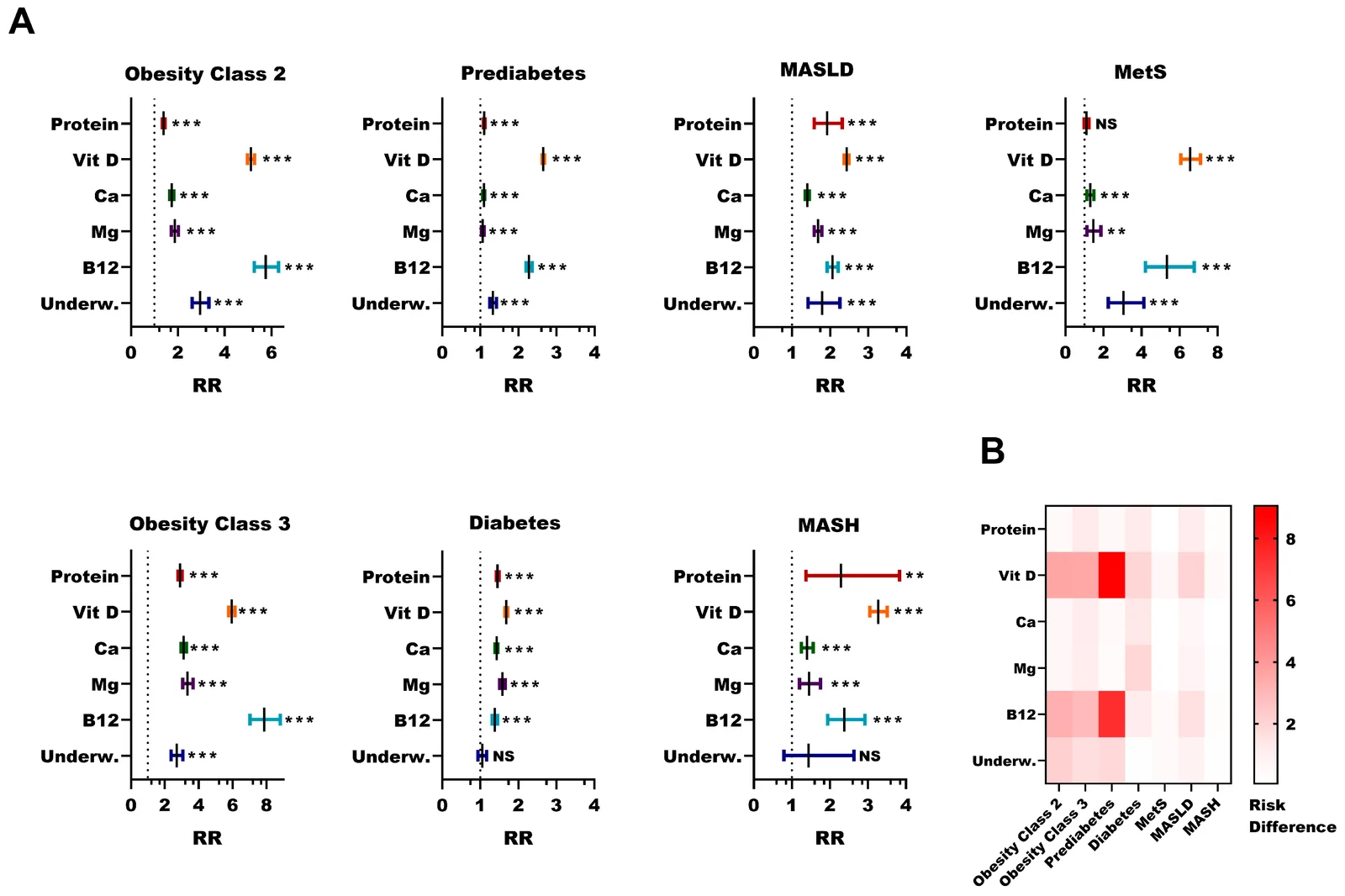
Obesity with deficiencies in vitamin D or B12 or previous underweight was more strongly associated with increased risk ratio of metabolic diseases compared to obesity without nutritional deficiencies. (**A**) Forest plot of risk ratio (RR) of metabolic outcomes in patients with obesity and nutritional deficiencies vs. the reference group. RR and confidence interval (CI) 95%. Significance: ** *p* < 0.01; *** *p* < 0.001; NS, non-significant. (**B**) Risk difference in patients with obesity and utritional deficiencies represented as a heat map. Risk difference equals the difference between the risk of nutritional deficiency and the risk in the obesity reference group. Scale: risk difference from 0 (white) to 9% (red). Risk differences: 1% weak (1 case per 100 exposed), 5% clinically significant (5 cases per 100 exposed), and 10% strong (10 cases per 100 exposed).

We found that vitamin D and vitamin B12 deficiencies showed the strongest associations with metabolic diseases, with increased risks of obesity class 2 (RR 5.12 and RR 5.75), obesity class 3 (RR 5.95 and RR 7.86), prediabetes (RR 2.65 and RR 2.28), metabolic syndrome (RR 6.55 and RR 5.33), MASLD (RR 1.92 and RR 2.06), and MASH (RR 3.27 and RR 2.38) (Table 4, Figure 8A). Calcium and magnesium deficiencies were associated with modest but significant increases in RR across most outcomes (Table 4, Figure 8A). However, despite achieving statistical significance, the risk differences were less than 1%, suggesting limited clinical significance (Figure 8B, Table 4).

#### 3.5.1. Severe Obesity

**Figure 9:**
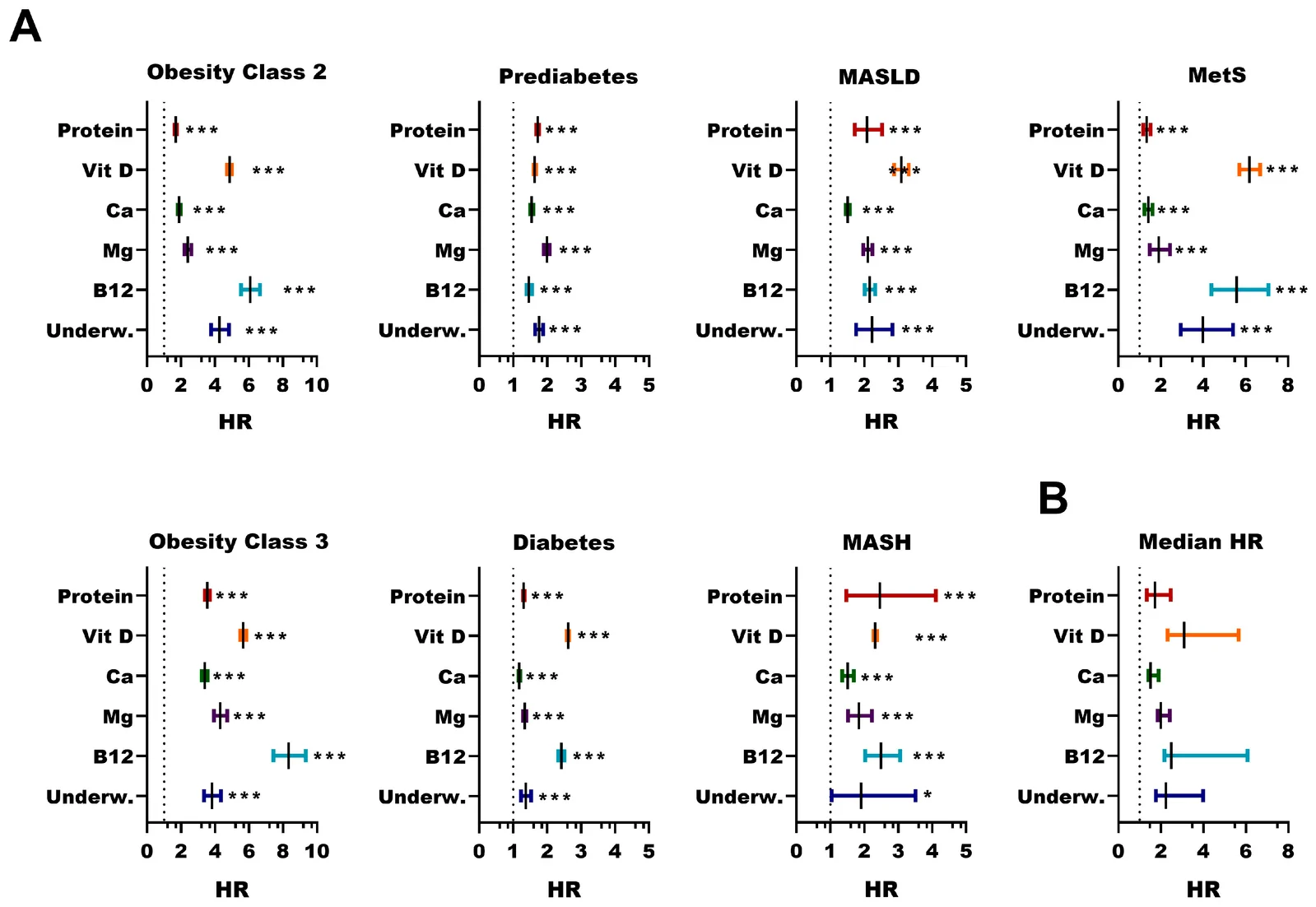
Obesity with deficiencies in vitamin D or B12 or previous underweight was associated with higher hazard ratio of developing metabolic diseases compared with obesity without nutritional deficiencies. (**A**) Forest plot of hazard ratio (HR) of metabolic outcomes in patients with obesity and nutritional deficiencies vs. the reference group. HR and confidence interval (CI) 95%. Significance: * *p* < 0.05, *** *p* < 0.001. (**B**) Forest plot of median HR and interquartile range (IQR) calculated from the seven HR values for each deficiency.

Severe obesity class 2 and class 3 had the strongest association with B12 deficiency (HR 6.08 and HR 8.33) followed by vitamin D deficiency (HR 4.87 and HR 5.66), whereas previous underweight status was more strongly associated with obesity class 2 than class 3 (HR 4.27 and HR 3.82) (Figure 9A, Table 5).

#### 3.5.2. Prediabetes/Diabetes 

Vitamin D deficiency (HR 2.62), vitamin B12 deficiency (HR 2.42), and previous underweight (HR 1.37) were more strongly associated with diabetes (Figure 9, Table 5) and also exhibited the greatest absolute risk differences for prediabetes (Table 4).

#### 3.5.3. MASLD/MASH

All nutritional deficiencies were associated with increased MASLD risk with the strongest associations for deficiencies in vitamin D (HR 3.09) and vitamin B12 (HR 2.16), as well as previous underweight (HR 2.23) (Table 5, Figure 8A), whereas MASH was most strongly associated with deficiencies in vitamin D (HR 2.32) and B12 (HR 2.49). Although effect estimates for deficiencies in calcium, magnesium, and protein increased after BMI adjustment (Tables S8 and S9), the absolute risk differences remained below 1% (Table 4, Figure 8B), suggesting limited clinical relevance despite significant associations.

#### 3.5.4. Metabolic Syndrome

The nutritional deficiencies analyzed here were associated with a significant risk of developing MetS components in patients with obesity (Table S7). Associations with MetS diagnosis were generally stronger when BMI was used for propensity score matching, compared with the initial obesity severity-based model (Tables S8 and S9). Deficiencies in vitamin D (HR 6.17), vitamin B12 (HR 5.57) and previous underweight (HR 3.98) were associated with a higher risk of developing MetS components, consistent with an even higher risk of MetS diagnosis (Table S7). Nevertheless, the absolute risk differences remained below 1% (Table 4, Figure 8B), suggesting limited clinical relevance despite these statistically significant associations.

#### 3.5.5. Time-to-Event Analysis

**Figure 10:**
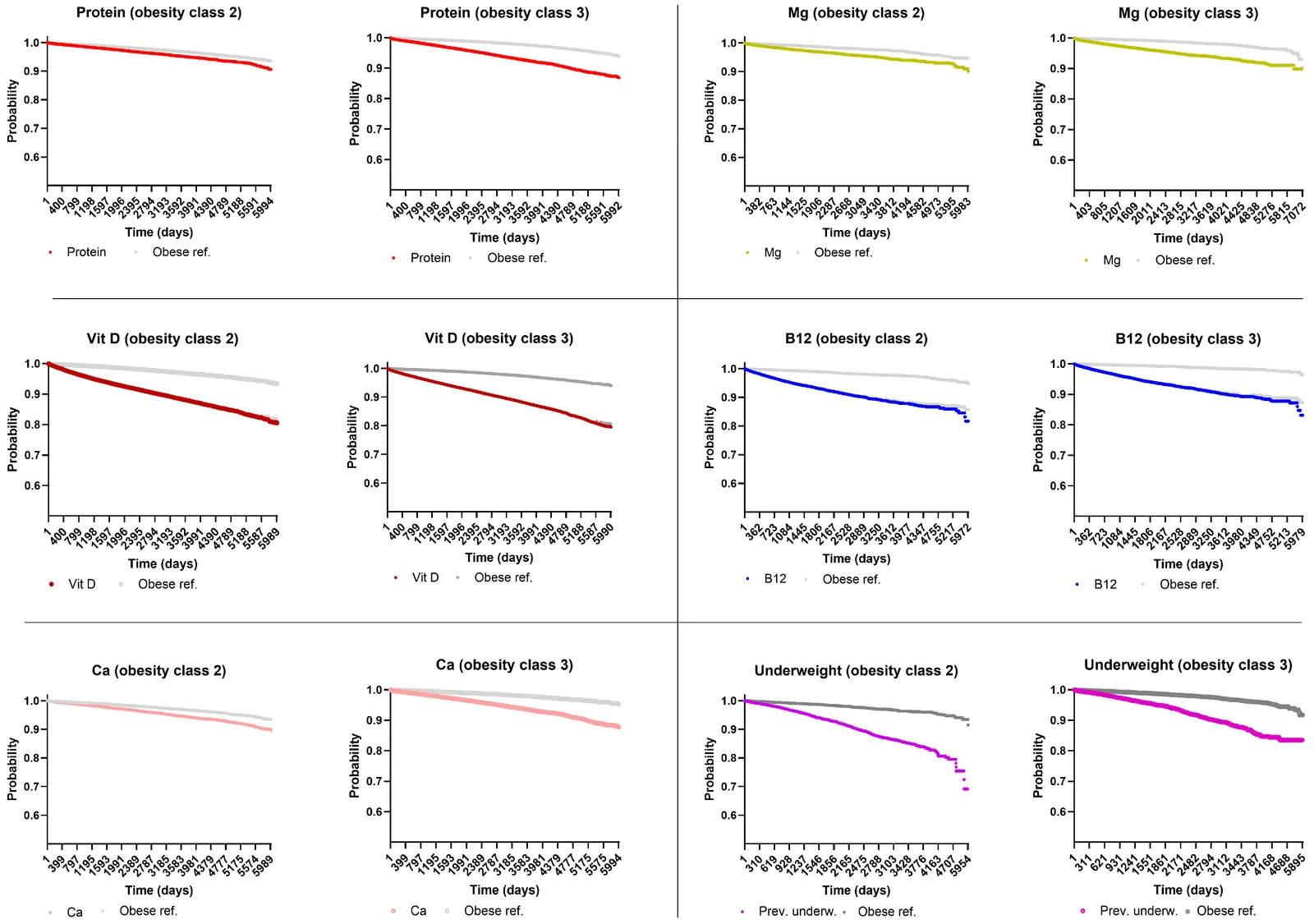
Obesity with deficiencies in vitamins D or B12 or previous underweight was more strongly associated with a risk of progression to severe obesity compared to obesity without nutritional deficiencies. Kaplan–Meier curves showing survival and the cumulative probability of diagnosis of severe obesity (class 2 or class 3) over time, in days from the index event, defined as the first diagnosis of obesity. Survival 1 represents no event; survival 0 represents that the event occurred in all patients.

**Figure 11:**
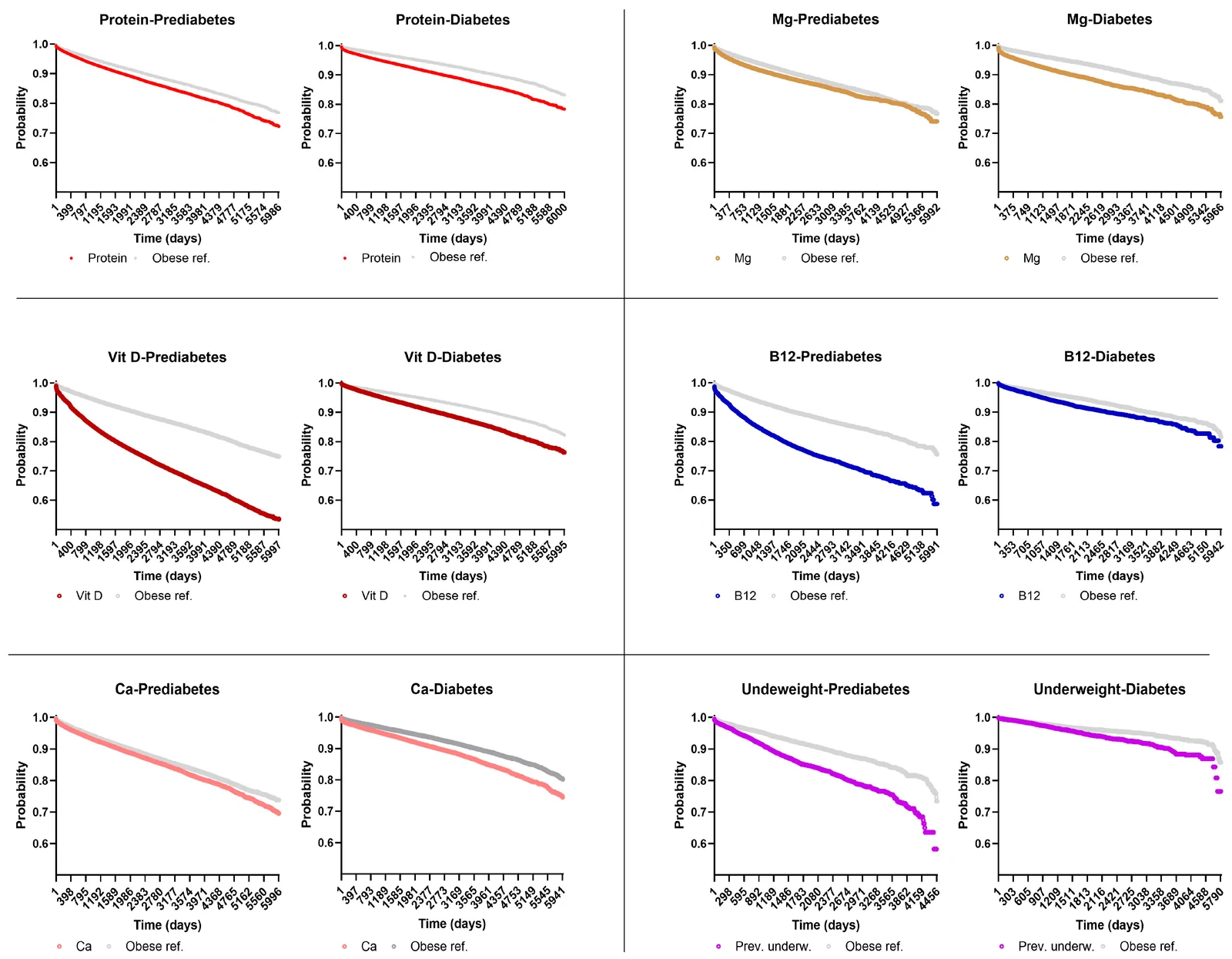
Obesity with deficiencies in vitamin D or B12 or previous underweight was more strongly associated with a risk of progression to prediabetes compared to obesity without nutritional deficiencies. Kaplan–Meier curves showing survival and the cumulative probability of diagnosis of prediabetes or diabetes over time, in days from the index event, defined as the first diagnosis of obesity. Survival 1 represents no event; survival 0 represents that the event occurred in all patients.

Time-to-event analyses using Kaplan–Meier curves showed a higher cumulative probability of progression to severe forms of obesity (classes 2 and 3) among patients with nutritional deficiencies than among those without (Figure 10). However, Kaplan–Meier curves among patients with calcium, magnesium, and protein deficiency showed limited curve separation across obesity, glycemic, and liver-related outcomes (Figure 10 and Figure 11 and Figure S2). In contrast, Kaplan–Meier curves among patients with vitamin D deficiency, vitamin B12 deficiency, and prior underweight status showed a higher cumulative probability of prediabetes than diabetes (Figure 11) and of MASLD than MASH (Figure S2). Suggesting that these nutritional deficiencies are more strongly associated with early metabolic dysfunction than with later-stage disease.

Overall, vitamin D deficiency (median HR 3.09, IQR 2.32–5.66), vitamin B12 deficiency (median HR 2.49, IQR 2.16–6.08), and prior underweight status (median HR 2.23, IQR 1.76–3.98) demonstrated the strongest associations across metabolic disease outcomes, whereas magnesium deficiency (median HR 1.99, IQR 1.84–2.41), protein deficiency (median HR 1.72, IQR 1.33–2.46), and calcium deficiency (median HR 1.51, IQR 1.41–1.89) were associated with comparatively lower risks (Figure 9B).

## 4. Discussion

As the global burden of obesity continues to increase worldwide, nutritional deficiencies are increasingly recognized as a prevalent, clinically relevant, yet often overlooked, aspect of obesity. Although the double burden of malnutrition is a major public health challenge in low- and middle-income countries, our analysis utilizing electronic healthcare records supports the growing body of evidence that nutritional deficiencies, principally vitamin D and vitamin B12 deficiencies, as well as a history of underweight, contribute to the metabolic vulnerability among individuals with obesity. In addition, the presence of hypertension at time of first obesity diagnosis emerged as a key predictor of adverse metabolic comorbidity outcomes, underscoring the importance of hypertension control in this population [35]. We further identified distinctive patterns of nutritional deficiencies in obesity and compared these characteristics with those observed in the general population irrespective of obesity status.

The higher prevalence of protein, vitamin B12, calcium, and magnesium deficiencies among U.S. patients with obesity likely reflects a lack of nutrient-dense foods such as meat, dairy, fruits, and vegetables [36]. The replacement of whole foods with energy-dense, ultra-processed products, that are low in essential nutrients, has been implicated in the presence of micronutrient deficiencies among individuals with obesity [36,37,38]. In addition, components of ultra-processed foods, such as microplastics and additives, can compromise the gut microbiome, promote chronic inflammation, and interfere with endocrine signaling, potentially contributing to the development of micronutrient deficiencies [37,39,40,41]. This “hidden hunger” of DBM differs in part from the observed in the general population, where micronutrient deficiencies are commonly driven by food insecurity, especially among those who suffer from racial and socioeconomic disparities [38,42]. However, the DBM may be driven by factors beyond food availability, including limited nutritional literacy and the preference for frequent consumption of nutrient-poor highly palatable ultra-processed foods. The protein leverage hypothesis suggests that unbalanced diets, diluted in essential nutrients, mainly protein, induce increased food intake as the individual attempt to satisfy unmet nutrient requirements, resulting in excess caloric consumption [43,44]. Studies also suggest that habitual consumption of ultra-processed foods dysrupt the appetite–satiety homeostasis [45]. These effects have been linked to alterations of the reward-related neural circuitry and dopamine-mediated food reward signaling, which increase the reinforcing value of highly palatable foods [46] and promote maladaptive eating behaviors, including emotional eating, stress eating, hedonic overeating, and addiction-like compulsive eating [45,46,47,48,49]. Understanding these mechanisms could help to explain why obesity and nutritional deficiencies frequently coexist and inform the development of targeted interventions to reduce the burden of malnutrition in modern societies.

Obesity with nutritional deficiencies peaked in middle-aged white females (mean age 46.8 years), highlighting midlife as a period of vulnerability. An age-related increase in adiposity in both sexes, beginning in the fourth decade of life [50], is associated with declining estrogen in perimenopause [51], and declining testosterone levels in men [52], combined with reduced physical activity and reduced total energy expenditure in middle age [51,53]. In women, enhanced baseline body fat, along with biological, sociocultural, and genetic factors, leads to greater weight gain than in men, who typically have a relatively higher baseline muscle mass [54]. In contrast, nutritional deficiencies in the general population more commonly affect children under 5 years of age and food-insecure adolescents [55,56]. Thus, our study suggests that the midlife metabolic transition is a key driver of the DBM in the high-income setting.

In contrast, older adults with obesity (>70 years) showed a high incidence of protein, calcium, magnesium, and vitamin B12 deficiencies, reflecting age-related changes such as reduced intestinal absorption [57,58] and declining renal function. Reduced conversion of vitamin D to its active form, calcitriol [59], further limits calcium absorption, while concomitant magnesium deficiency impairs both parathyroid hormone secretion and vitamin D metabolism [60]. Inflammaging, triggered by amino acids and micronutrient deficiencies [61,62,63], frailty, and social determinants further increase the incidence of nutritional deficiencies in older adults [56,64].

The high burden of nutritional deficiencies among Black/African American patients with obesity aligns with their geographical concentration in the U.S. South [65], a region characterized by lower dietary quality, reduced dietary supplement use, and limited access to fresh foods [66,67,68,69]. Although the Southern regions have a high ultraviolet B (UVB) index, which is needed for the production of vitamin D in the skin, the presence of skin melanin, which reduces the efficiency of the conversion of 7-dehydrocholesterol to previtamin D_3_, could lead to vitamin D deficiency in these communities [70,71]. These findings highlight that addressing vitamin D deficiency requires a multifaceted approach, including support of vitamin D metabolism in addition to adequate sun exposure and nutritional supplementation.

Obese patients with a history of underweight had one of the highest cumulative burdens of metabolic diseases. Studies suggest that the transition from early nutritional deprivation to later caloric abundance creates a metabolic mismatch that places stress on an already constrained physiological capacity and predisposes individuals to metabolic diseases [72,73,74]. These findings underscore the enduring effects of early nutritional programming on metabolic health and disease risk later in life.

The low prevalence of a history of underweight among individuals with obesity (1.9%) found in this study is consistent with U.S. population estimates of approximately 2% [75,76]. Although relatively uncommon, this proportion corresponds to an estimated 6.8 million individuals in the U.S. population who may be at elevated metabolic risk among 340 million people [77]. Overall, our findings suggest that both weight history and nutritional status are important indicators of metabolic disease risk, highlighting the public health significance of this group.

In contrast to the general Hispanic population [42], our finding showed that Hispanics with obesity were not likely to have nutritional deficiencies, possibly due to the relatively high nutritional quality of their dietary intake [78,79,80]. The most notable difference in this group was the higher prevalence and incidence of previous underweight, which may reflect the higher rates of food insecurity among Hispanics in U.S. [16,81].

Nevertheless, the clustering of patients with obesity and a previous history of underweight in the East North Central region is unlikely to be explained solely by food insecurity, as this region does not exhibit the highest rates of food insecurity [82]. Nor is the pattern likely attributable to urbanization alone, given the elevated prevalence of obesity observed in both small metropolitan and rural populations [83]. Instead, this geographical concentration may reflect rural health disparities [84], and/or a more comprehensive diagnosis documentation within healthcare systems [85], combined with the fact that East North Central States are part of the Midwest, the U.S. region with the highest adult obesity prevalence [7].

A greater prevalence of vitamin B12 deficiency among white patients with obesity was unexpected, given their relatively low rates of food insecurity [42] and previous research showing an inverse association between food insecurity and vitamin B12 status [86]. Metformin therapy is a recognized risk factor for vitamin B12 deficiency [87]; however, metformin use was included as covariate in our analysis. One possible explanation could be related to higher rates of alcohol consumption reported in the white non-Hispanic population [88], as chronic alcohol use can impair vitamin B12 absorption and contribute to deficiency. Alternatively, vitamin B12 deficiency may be associated with dietary patterns characterized by lower consumption of animal-source foods [89] in subgroups of the white population, as is true for Asian patients. However, the reasons underlying the higher prevalence of vitamin B12 deficiency among white patients with obesity remain to be elucidated through further research. Given that vitamin B12 deficiency had the second-highest cumulative burden of metabolic diseases in our study, it suggests that B12 supplementation may mitigate metabolic risks.

All nutritional deficiencies increased the burden of individual MetS components in individuals with obesity, but only vitamin D, vitamin B12, and prior underweight translated into clinically recognized MetS in the initial PSM model, consistent with findings in the general population [90,91,92]. Protein, calcium and magnesium deficiencies have been linked to metabolic dysfunction [93,94,95,96], yet their association with MetS was weak in this study and become apparent only after PSM for BMI. Moreover, absolute risk differences of less than 1% indicate that, despite statistical significance, the clinical impact of these deficiencies on MetS risk may be limited.

Micronutrient deficiencies included in this study were associated with an increased risk for MASLD, suggesting their role in disease onset. The effects of vitamin D deficiency on insulin resistance, low-grade systemic inflammation, and fibrogenesis [63] are well established, and a history of underweight may promote steatosis via alterations in lipid metabolism, the gut microbiome, and intestinal absorption [72,97,98]. Protein, calcium, and magnesium deficiencies were associated with MASLD but not with the more severe form of disease, MASH, suggesting either that these deficiencies play a greater role in the development of early steatosis, or that early clinical recognition, and subsequent nutritional or medical interventions, may have reduced the likelihood of progression to MASH in these individuals.

In contrast to studies reporting a direct association between higher serum vitamin B12 levels and MASLD [99,100], our findings demonstrated an inverse association and are consistent with studies linking vitamin B12 deficiency to visceral adiposity, insulin resistance, and hepatic steatosis [101]. This discrepancy suggests that obesity-related mechanisms, such as protection from oxidative stress and inflammation [102,103], may modify the relationship between vitamin B12 status and steatotic liver disease. Nevertheless, our findings suggest that vitamin B12 deficiency, vitamin D deficiency, and a history of underweight are more strongly associated with early metabolic dysregulation, as reflected by the greater separation of Kaplan–Meier curves for MASLD, prediabetes, and obesity class 2 than for MASH, diabetes and obesity class 3.

As protein, vitamin D, calcium, and magnesium are important for bone mineralization and muscle function [104], deficiencies in these nutrients indicate adverse effects on musculoskeletal health for individuals experiencing these types of DBM. Obesity and undernutrition increase the risk of osteosarcopenia in adults >50 years [105,106], likely through inflammatory pathways, low-grade inflammation, impaired glucose homeostasis, and dysregulated adipokine milieu [107]. Our findings suggest that the nutritional deficiencies common in the U.S. may also accelerate the progression toward osteosarcopenic obesity [106,108,109].

Our data indicated a 21.3% prevalence of obesity without nutritional deficiencies, which is lower than the Centers for Disease Control and Prevention (CDC) estimates for the general adult population in 2024, where obesity prevalence exceeded 25%, and was more than 30% in most jurisdictions [7]. However, this comparison is not equivalent, as the CDC estimates reflect obesity prevalence regardless of nutritional deficiency status. Our data may also underestimate prevalence, as they are derived from healthcare-seeking populations and require a documented BMI and diagnosis of obesity [110]. Our risk estimates may also be conservative due to stringent exclusion criteria, including chronic inflammatory conditions, malabsorption, bariatric surgery, pregnancy, and coexisting deficiencies. These exclusions improved the internal validity of the metabolic outcome analysis; however, they precluded an assessment of those conditions. Similarly, iron deficiency may be underdiagnosed, as it is often clinically recognized when it progresses to overt anemia [111]. Furthermore, the exclusion of major confounding conditions, such as chronic kidney disease, endocrine disorders, and other secondary causes of malnutrition, may have reduced the identification of individuals with iron deficiency and other nutritional deficiencies, potentially leading to an underestimation of the prevalence. In addition, patients with multiple concurrent nutritional deficiencies were excluded to improve the specificity of the outcome analysis and to better isolate the associations attributable to individual deficiencies. Despite these limitations, a low incidence of deficiencies in zinc, folate, and vitamin A in DBM in the U.S. relative to global malnutrition patterns [112] likely reflects the positive impact of long-standing public health measures, such as folic acid fortification of grains and vitamin A enrichment of foods, including ultra-processed products [113]. The results suggest that obesity-related nutritional deficiencies are shaped not only by food intake and physio-metabolic determinants but also by government policies related to food nutrition.

## 5. Conclusions

In conclusion, this study indicates that vitamin D and vitamin B12 deficiencies, as well as a history of underweight, are relevant drivers of adverse long-term metabolic outcomes in patients with obesity and warrant prioritization in future research. Addressing these factors should be part of personalized medicine strategies aimed at improving metabolic health.

## Figures and Tables

**Table 1:** Definitions and inclusion criteria for nutritional deficiencies at first diagnosis of obesity.

Protein Deficiency	Levels in Serum	TriNetX Code
Severe protein-calorie malnutrition	n/a	E43
Moderate protein-calorie malnutrition	n/a	E44.0
Albumin (with CRP < 10 mg/L) *	<3.5 g/dL	TNX curated (9045); LOINC (1751-7)
Protein (with CRP < 10 mg/L)	<6.0 g/dL	TNX curated (9053); LOINC 2885-2
Prealbumin with CRP < 10 mg/L	<16 mg/dL	TNX curated (LG2038-0)
**Micronutrient Deficiency**		
Vitamin D deficiency	<20 ng/mL	E55, TNX CURATED (LG25965-1)
Dietary Iron deficiency Ferritin with CRP < 10 mg/L	<15 ng/mL	IDC-9-CM: 280.1, TNX CURATED (9042), ICD-10 (E61.1)
Dietary Vitamin B12 Deficiency	<200 pg/mL	D51.3, TNX CURATED (9065)
Dietary Zinc Deficiency	<0.7 μg/mL	E60, TNX CURATED (LG23959-6)
Dietary Calcium deficiency **	<8.5 mg/dL	E58, TNX CURATED (9022)
Magnesium Deficiency	<1.7 mg/dL	E61.2, TNX CURATED (9026)
Folate	<2 ng/mL	D52.0, TNX CURATED (9067)
Vitamin A deficiency, Retinol with CRP < 10 mg/L	<0.2 μg/mL	E50, TNX CURATED (LG11471-6)
**Underweight > 6 mo Before**		
Underweight	n/a	R63.6
BMI < 18.5 kg/m^2^ > 18 Y	n/a	9093
BMI pediatric, less than 5th percentile for age	n/a	Z68.51
Kwashiorkor, Marasmus, Marasmic Kwashiorkor	n/a	E40, E41, E42

* To minimize misclassification of protein deficiency, patients with CRP > 10 mg/L and patients with chronic kidney disease were excluded from the study. ** Total serum calcium may result in exposure misclassification in individuals with altered protein status.

**Table 2:** Incidence and prevalence trends in patients at first diagnosis of obesity with nutritional deficiencies. The reference group are patients at first diagnosis of obesity without a history of nutritional deficiency. Source: electronic health records, TriNetX Research Network, year 2025.

Deficiency	Incidence (%)	Incident Cases	Prevalence (%)	Prevalent Cases
Protein	1.9	77,032	8.9	384,614
Vit D	2.1	85,648	10.6	472,843
Ca	1.5	63,312	7.5	335,511
Mg	0.6	28,379	2.5	113,055
B12	0.1	4007	0.5	21,236
Underweight *	0.3	819	1.9	6041
Obese reference	5	917,360	21.3	4,718,439

* Underweight status occurred more than 6 months before the first obesity diagnosis. Obese reference without history of nutritional deficiency.

**Table 3:** Number of patients at first diagnosis of obesity per nutritional deficiency compared with a reference cohort of patients with obesity without a history of nutritional deficiency.

Region	Protein	Vit D	Ca	Mg	B12	Underweight	Total	Obese Ref.
South Atlantic	157,304	154,912	101,783	39,475	18,954	4062	476,490	729,449
Middle Atlantic	67,793	89,248	80,094	39,414	18,049	10,240	304,838	2,336,949
West South Central	77,235	84,334	51,045	13,551	7850	1005	235,020	909,203
East North Central	12,101	75,900	16,482	5055	7892	10,546	127,976	917,287
West North Central	29,027	51,548	19,960	4764	5527	626	111,452	3,216,105
Mountain	13,564	42,746	28,980	5471	3946	950	95,657	411,948
New England	12,320	50,272	14,478	3714	3521	2802	87,107	1,829,967
East South Central	17,543	17,713	22,339	14,017	5621	811	78,044	1,020,476
Pacific	15,591	11,168	15,923	3958	1241	283	48,164	517,367
Ex-US	1364	5415	1705	1759	341	106	10,690	549,080
Total	403,842	583,256	352,789	131,178	72,942	31,431	1,575,438	12,437,831

**Table 4:** Risk of metabolic diseases in patients with obesity and nutritional deficiencies compared with a reference cohort of patients with obesity without a history of nutritional deficiency.

Condition	Deficiency	Risk Exposed (%)	Risk Obese (%)	RD	RR (95% CI)	OR (95% CI)	*p*
Obesity Class 2	Protein	1.26	0.9	0.35%	1.39 (1.34–1.45)	1.40 (1.34–1.45)	<0.001
	Vit D	4.5	0.88	3.62%	5.12 (4.98–5.28)	5.32 (5.16–5.48)	<0.001
	Ca	1.49	0.86	0.63%	1.74 (1.66–1.82)	1.75 (1.67–1.83)	<0.001
	Mg	1.4	0.75	0.65%	1.87 (1.73–2.03)	1.88 (1.74–2.04)	<0.001
	B12	3.98	0.69	3.29%	5.75 (5.26–6.30)	5.95 (5.43–6.52)	<0.001
	Underweight	3.27	1.11	2.16%	2.95 (2.61–3.33)	3.01 (2.66–3.41)	<0.001
Obesity Class 3	Protein	1.93	0.66	1.26%	2.90 (2.78–3.02)	2.93 (2.82–3.06)	<0.001
	Vit D	4.24	0.71	3.53%	5.95 (5.76–6.14)	6.17 (5.97–6.37)	<0.001
	Ca	1.8	0.58	1.22%	3.11 (2.95–3.27)	3.15 (2.99–3.31)	<0.001
	Mg	1.65	0.49	1.16%	3.34 (3.05–3.66)	3.38 (3.08–3.70)	<0.001
	Vit B12	3.32	0.42	2.89%	7.86 (7.02–8.81)	8.10 (7.22–9.08)	<0.001
	Underweight	2.68	0.99	1.69%	2.70 (2.37–3.07)	2.75 (2.41–3.13)	<0.001
Prediabetes	Protein	5.35	4.85	0.50%	1.10 (1.08–1.12)	1.11 (1.09–1.13)	<0.001
	Vit D	14.55	5.49	9.06%	2.65 (2.62–2.68)	2.93 (2.89–2.97)	<0.001
	Ca	6.19	5.7	0.49%	1.09 (1.06–1.11)	1.09 (1.07–1.12)	<0.001
	Mg	5.47	5.15	0.33%	1.06 (1.03–1.10)	1.07 (1.03–1.11)	0.0006
	B12	13.14	5.77	7.37%	2.28 (2.20–2.36)	2.47 (2.38–2.57)	<0.001
	Underweight	7.18	5.38	1.80%	1.33 (1.25–1.42)	1.36 (1.27–1.45)	<0.001
Diabetes	Protein	4.06	2.79	1.26%	1.45 (1.42–1.49)	1.47 (1.44–1.51)	<0.001
	Vit D	4.85	2.88	1.97%	1.68 (1.65–1.72)	1.72 (1.68–1.75)	<0.001
	Ca	4.57	3.21	1.36%	1.43 (1.39–1.46)	1.45 (1.41–1.48)	<0.001
	Mg	5.19	3.29	1.90%	1.58 (1.51–1.65)	1.61 (1.54–1.68)	<0.001
	B12	4.44	3.22	1.22%	1.38 (1.31–1.45)	1.40 (1.32–1.47)	<0.001
	Underweight	2.39	2.28	0.11%	1.05 (0.94–1.17)	1.05 (0.94–1.17)	0.4
MetS	Protein	0.115	0.105	0.01%	1.10 (0.98–1.24)	1.10 (0.98–1.24)	0.121
	Vit D	0.72	0.11	0.61%	6.55 (6.06–7.09)	6.59 (6.09–7.14)	<0.001
	Ca	0.145	0.111	0.03%	1.30 (1.14–1.49)	1.30 (1.14–1.49)	<0.001
	Mg	0.127	0.088	0.04%	1.46 (1.14–1.86)	1.46 (1.14–1.86)	0.0028
	B12	0.534	0.1	0.43%	5.33 (4.20–6.76)	5.35 (4.21–6.80)	<0.001
	Underweight	0.56	0.18	0.38	3.05 (2.26–4.12)	3.06 (2.27–4.13)	<0.0001
MASLD	Protein	2.55	1.33	1.22%	1.92 (1.58–2.32)	1.94 (1.60–2.36)	<0.001
	Vit D	3.43	1.41	2.02%	2.43 (2.37–2.49)	2.48 (2.42–2.55)	<0.001
	Ca	2.1	1.5	0.60%	1.40 (1.35–1.45)	1.41 (1.36–1.46)	<0.001
	Mg	2.26	1.35	0.91%	1.68 (1.58–1.78)	1.69 (1.59–1.80)	<0.001
	B12	3.07	1.49	1.58%	2.06 (1.92–2.21)	2.09 (1.95–2.25)	<0.001
	Underweight	2.27	1.26	1.00%	1.79 (1.42–2.26)	1.81 (1.43–2.29)	<0.001
MASH	Protein	0.39	0.17	0.22%	2.29 (1.37–3.83)	2.30 (1.38–3.84)	0.0011
	Vit. D	0.52	0.16	0.36%	3.27 (3.05–3.51)	3.28 (3.06–3.52)	<0.001
	Ca	0.22	0.16	0.06%	1.40 (1.25–1.56)	1.40 (1.25–1.57)	<0.001
	Mg	0.21	0.15	0.07%	1.45 (1.20–1.75)	1.45 (1.20–1.75)	<0.001
	B12	0.39	0.16	0.23%	2.38 (1.94–2.92)	2.39 (1.95–2.93)	<0.001
	Underweight	0.3	~0.21	0.09%	1.44 (0.79–2.63)	1.40 (0.98–1.99)	0.22

RR and OR >1 indicate an increased risk associated with nutritional deficiency, whereas values <1 indicate a decreased risk. In large cohorts, risk differences (RD) <1% may have limited clinical relevance despite statistical significance.

**Table 5:** Hazard ratio (HR) comparing the relative risk over time of developing a more severe metabolic outcome in patients after first diagnosis of obesity and nutritional deficiencies compared with the patients with obesity without a history of nutritional deficiency.

Deficiency	Outcome Category	N Patients	Events	HR	95% CI	*p*-Value
Protein	Obesity Class 2	476,310	5980	1.69	1.63–1.76	<0.001
	Obesity Class 3	473,461	9119	3.55	3.41–3.69	<0.001
	Prediabetes	419,258	17,012	1.72	1.68–1.76	<0.001
	Diabetes	441,094	23,600	1.31	1.29–1.34	<0.001
	MetS	481,621	554	1.33	1.18–1.51	<0.001
	MASH	12,240	48	2.46	1.47–4.11	<0.001
	MASLD	11,502	293	2.08	1.72–2.52	<0.001
Vit D	Obesity Class 2	576,786	25,968	4.87	4.72–5.01	<0.001
	Obesity Class 3	574,076	24,350	5.66	5.48–5.85	<0.001
	Prediabetes	521,621	25,297	1.63	1.60–1.67	<0.001
	Diabetes	473,480	68,894	2.62	2.58–2.65	<0.001
	MetS	639,674	4606	6.17	5.70–6.68	<0.001
	MASH	622,576	21,376	2.32	2.27–2.38	<0.001
	MASLD	644,668	3337	3.09	2.88–3.31	<0.001
Ca	Obesity Class 2	329,786	4926	1.89	1.80–1.98	<0.001
	Obesity Class 3	328,532	5926	3.39	3.22–3.57	<0.001
	Prediabetes	282,049	12,897	1.54	1.50–1.59	<0.001
	Diabetes	303,220	18,759	1.18	1.16–1.21	<0.001
	MetS	333,783	483	1.41	1.23–1.61	<0.001
	MASH	332,900	730	1.51	1.35–1.69	<0.001
	MASLD	321,954	6766	1.51	1.46–1.57	<0.001
Mg	Obesity Class 2	119,311	1667	2.41	2.22–2.62	<0.001
	Obesity Class 3	118,821	1962	4.31	3.93–4.72	<0.001
	Prediabetes	90,474	4698	1.99	1.90–2.07	<0.001
	Diabetes	109,694	6004	1.34	1.29–1.39	<0.001
	MetS	120,903	154	1.9	1.48–2.43	<0.001
	MASH	119,908	257	1.84	1.52–2.23	<0.001
	MASLD	114,335	2580	2.1	1.97–2.24	<0.001
B12	Obesity Class 2	74,732	2975	6.08	5.55–6.66	<0.001
	Obesity Class 3	74,913	2484	8.33	7.43–9.34	<0.001
	Prediabetes	64,588	2868	1.46	1.39–1.55	<0.001
	Diabetes	61,090	8027	2.42	2.33–2.51	<0.001
	MetS	78,987	422	5.57	4.38–7.07	<0.001
	MASH	79,562	311	2.49	2.03–3.06	<0.001
	MASLD	76,125	2337	2.16	2.01–2.32	<0.001
Underweight	Obesity Class 2	30,498	998	4.27	3.78–4.83	<0.001
	Obesity Class 3	30,768	824	3.82	3.35–4.35	<0.001
	Prediabetes	27,373	1965	1.76	1.65–1.88	<0.001
	Diabetes	28,573	682	1.37	1.23–1.53	<0.001
	MetS	30,740	173	3.98	2.94–5.40	<0.001
	MASH	8615	26	1.91	1.04–3.51	0.033
	MASLD	8565	194	2.23	1.76–2.83	<0.001

## Data Availability

Data are available on the TriNetX Research Network to qualified institutions with an active TriNetX subscription.

## Supplementary Materials

The following supporting information can be downloaded at: https://www.mdpi.com/article/10.3390/nu18183005/s1, Figure S1: Laboratory values in patients with obesity and nutritional deficiencies vs. patients with obesity without history of nutritional deficiencies (obese ref); Figure S2: Kaplan–Meier curves showing the cumulative probability of diagnosis of MASLD or MASH over time among patients with obesity and nutritional deficiencies, compared with an obese reference group without nutritional deficiencies; Table S1: Inclusion criteria for diagnosis of Obesity*, number of eligible patients and TriNetX codes per category; Table S2: Exclusion criteria in cohorts of patients with confounding conditions, and TriNetX codes; Table S3: Exclusion Criteria of cofounding diseases or inflammatory conditions; Table S4: Metabolic outcomes studied and TriNetX codes; Table S5: Temporal Trends in incidence and prevalence of obesity with nutritional deficiencies, 2021–2025, electronic health records (TriNetX, Research Network); Table S6: Representative laboratory values associated with metabolic diseases in cohorts of patients with obesity and nutritional deficiencies and the reference obese cohort without a history of nutritional deficiency; Table S7: Risk of diagnosis of metabolic syndrome (MetS) and its components in patients with obesity and nutritional deficiencies compared with patients with obesity but no history of nutritional deficiency; Table S8: Sensitivity analyses show the risk ratio (RR) and 95% confidence interval (95% IC) of metabolic outcomes among patients with obesity and nutritional deficiencies relative to a reference cohort of patients with obesity without nutritional deficiencies; Table S9: Sensitivity analyses show the hazard ratio (HR) and 95% confidence interval (95% CI) of metabolic outcomes among patients with obesity and nutritional deficiencies relative to a reference cohort of patients with obesity without nutritional deficiencies.
