# Supplementary material for: Enhanced Metabolic Risks in Patients with Obesity and Nutritional Deficiencies in the U.S.: A TriNetX Study
Source: Nutrients. 2026 Sep 14;18(18):3005. doi: 10.3390/nu18183005 (PMC13610809; doi:10.3390/nu18183005)
Supplement: Supplementary file 1 [file nutrients-18-03005-s001.zip › nutrients-4534356-supplementary.pdf]

Figure S1. Laboratory values in patients with obesity and nutritional deficiencies vs. patients with obesity without history of nutritional deficiencies (obese ref). Data is the mean and SD, n=number of patients.****p<0.0001; ***p<0.001; **p<0.001, One-way Anova, Dunnett's multiple comparison test.

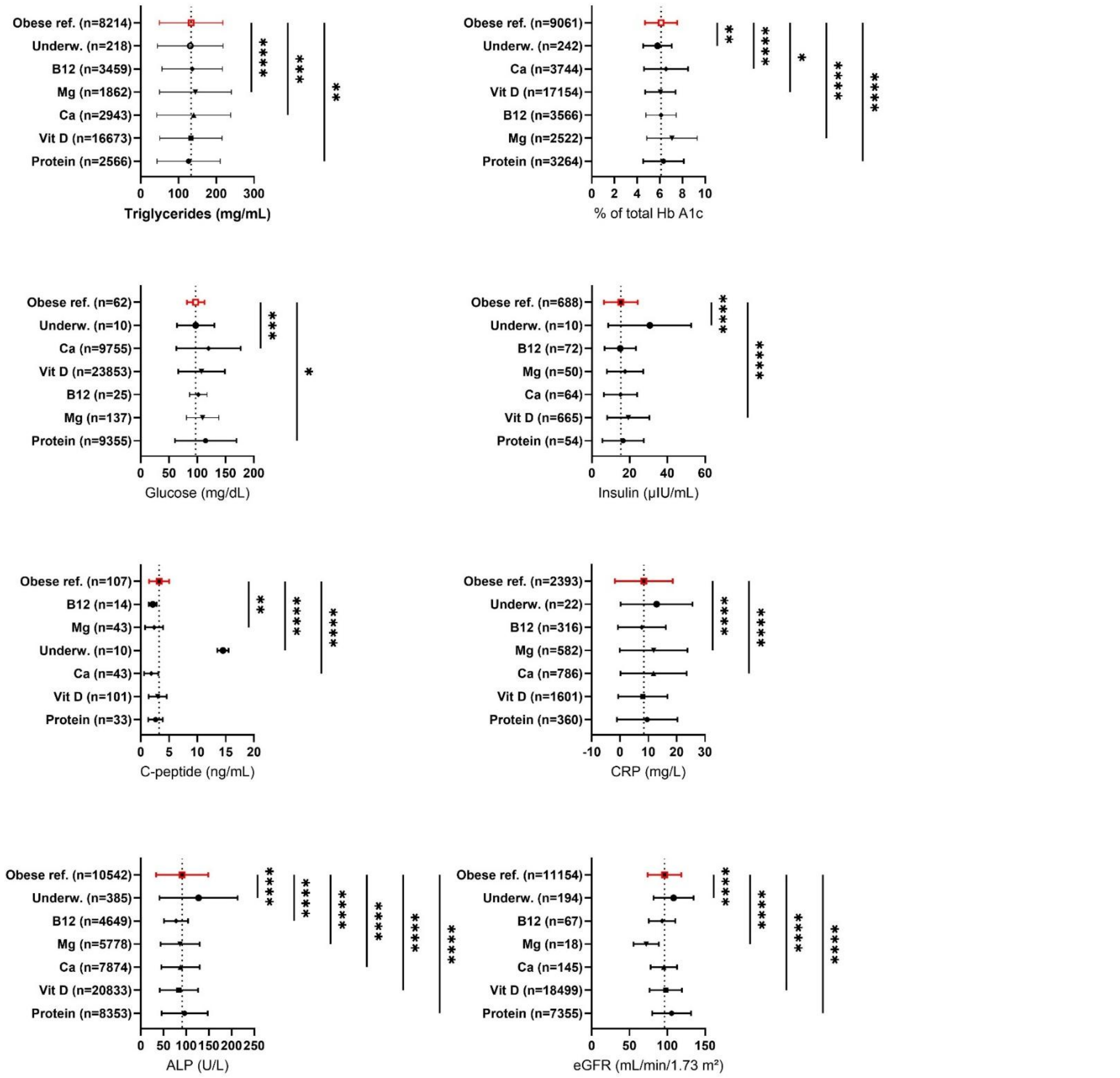

Figure S2. Kaplan–Meier curves showing the cumulative probability of diagnosis of MASLD or MASH over time among patients with obesity and nutritional deficiencies, compared with an obese reference group without nutritional deficiencies. Time is presented in days from the index event, defined as the first diagnosis of obesity. Survival 1 represents no event; survival 0 represents that all patients show the event. The outcome reflects incidence through the time, not mortality. Differences between groups were assessed using the log-rank test (**Table 5**).

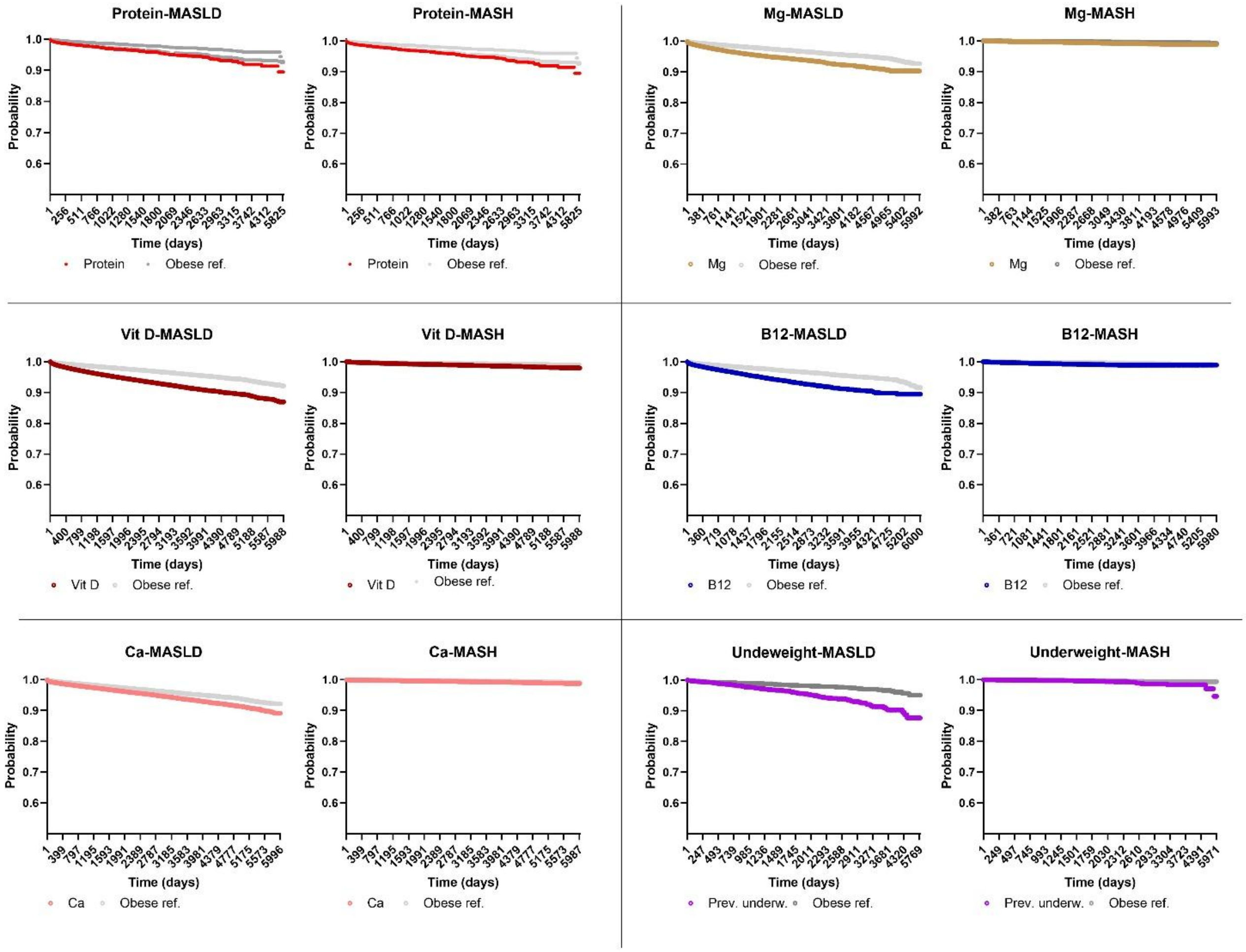

Table S1. Inclusion criteria for diagnosis of Obesity*, number of eligible patients and TriNetX codes per category.

Description	Code	Counts**
BMI >30 kg/m2 >18Y	LOINC: 9083	77,737,723
Obesity Unspecified	E66.9	9,030,027
Obesity due to excess calories	E66.0	5,916,971
Morbid (severe) obesity due to excess calories	E66.01	4,619,837
Other Obesity	E66.8	3,7,7,281
Obesity Class	E66.81	3,455,636
Obesity, class 1	E66.811	1,747.028
Obesity, class 2	E66.812	1,163,929
Obesity, class 3	E66.813	1,073,510
Other obesity not elsewhere classified	E66.89	55,950
Childhood obesity, class 1	Z68.54	1,086,466
Severe childhood obesity, class 2	Z68.55	54,410
Extremely severe childhood obesity, class 3	Z68.56	42,494

*No age range restricted.** A single patient could satisfy more than one inclusion criteria and could be counted in multiple inclusion categories. Therefore, the number of counts should not be summed up to estimate the total number of unique patients.

Table S2. Exclusion criteria in cohorts of patients with confounding conditions, and TriNetX codes.

**Description**	**Code**	**Counts***
Malignant Neoplasms, Neoplasm	C00-D49, C80.1	23,199,115
Transplants	Z94, Z94.8, Z94.9	785,974
Pregnancy	Z33	1,197,855
Congenital and genetical disorders	E84, Q80-Q89,Q65-Q79, Q90-Q99	4,025,303
Syndromic/Genetic abnormalities	Q87, E24	252,845
Drug induced Obesity	E66.1	62,038
Acute kidney failure and chronic kidney disease	N17-N19	7,984,552
Other chronic obstructive pulmonary disease	J44	4,052,824
Burns and corrosions	T20–T25, T30-T32, T26-T28	6,257,166
Poisoning by drugs	T36–T50	4,549,958
Toxic effects of substances	T51–T65	1,092,339
Hearth diseases	I20-I25, I30-I52, I50	30,391,203
Endocrine disorder (thyroid/pituitary gland)	E00-E07, E20-E23	11,218,254
Disorders of amino acid metabolism	E70, E71, E72	288,468
Intestinal malabsorption	K90	831,557
Bariatric surgery	Z98.84	668,968
Postsurgical malabsorption	K91.2	135,651
Dependence on renal dialysis	Z99.2	583,774
Cachexia	R64	434,368.
Starvation	T73.0	49,601
Bone marrow failure syndromes, hemolytic anemias	D55–D59, D61, D46	1,678,151
Acute posthemorrhagic anemia	D62	2,520,815
Fibrosis and cirrhosis of liver	K74	1,102,531

* Counts are not mutually exclusive, and a single patient may have contributed to more than one exclusion category. Therefore, the number of counts meeting each exclusion criteria should not be summed to estimate the total number of unique patients excluded.

Table S3. Exclusion Criteria of cofounding diseases or inflammatory conditions.

Description	Code
**Protein deficiency**	
ESR >40 mm/hr; CRP >10mg/mdL	TNX curated: 9066; 9063
Other nutritional deficiencies (vitamins and minerals)	E50–E64
**All Micronutrient deficiencies**	
protein-calorie malnutrition	E43–E46
**Vitamin D deficiency**	
CKD stage 4–5	N18.4, N18.5
Cirrhosis	K74.6
Malabsorption syndromes	K90, K91.2
Long-term corticosteroid therapy	Z79.52
Hyperparathyroidism	E21
**Vitamin B12 deficiency:** iron or folate deficiency	D50, D52, E61.0-E61.6
**Magnesium deficiency:** iron, folate, B12, vitamin D, or calcium deficiency	D50, D51, D52, D51.3, E58
**Calcium deficiency:** iron, folate, B12, vitamin D, or magnesium deficiency	D50, D51, D52, D51.3

Table S4. Metabolic outcomes studied and TriNetX codes.

Outcome	Code
**Severe Obesity**	
Obesity class 2	E66.812, Z68.55
Obesity class 3	E66.813; Z68.56
**Pre-Diabetes & Diabetes**	
Prediabetes & Hb1c 5.7–6.4%	R73.03; LOINC: 9037
Diabetes mellitus& Hb1c >6.5%& Fasting Glucose≥126 mg/dL	E08-E13, E11, LOINC: 9037, LOINC: 1558-6
**Metabolic Syndrome**	
**Metabolic syndrome Diagnosis**	E88.810
**Metabolic syndrome components:**	
Obesity* and two of the following:	
Hypertriglyceridemia: triglycerides ≥150 mg/dL or lipid-lowering therapeutics	TNX Curated 9004, C10AA, C10AB, C10AC, C10AD
HDL men 40 mg/dL- women 50mg/dL	TNX Curated 9001
Fasting glucose ≥126 mg/dL, H1c>6.5%, or diabetes Diagnosis or drugs used in diabetes	E11, LOINC: 1558-6; TNX:9037 (>6.5%), ATC:A10
Hypertension; anti-hypertensive agents	I10, ATC: C07, C08, C09, C02
**Non-Alcoholic Fatty Liver Disease****	
MASH/NASH	K75.81
Fatty liver/MASLD	K76.0
**Exclude alcoholic liver disease	K70

*Obesity definition was used as a surrogate for central obesity (waist circumference) when defining metabolic syndrome components. This adapted definition differs from standard Adult Treatment Panel and International Diabetes Federation criteria and may have resulted in some misclassification of metabolic syndrome status.

Table S5. Temporal Trends in incidence and prevalence of obesity with nutritional deficiencies, 2021–2025, electronic health records (TriNetX, Research Network).

Year	Incidence	Incidence	Incident	Prevalence	Prevalent
**Protein**					
**2021**	1.1	3.53E-05	68,954	5.2	332,304
**2022**	1.3	4.05E-05	76,291	5.9	365,178
**2023**	1.6	5.04E-05	88,755	6.7	395,866
**2024**	1.8	5.79E-05	88,717	7.7	410,199
**2025**	1.9	8.47E-05	77,032	8.9	384,614
**Vit D**					
**2021**	1.1	3.30E-05	66,702	4.8	314,601
**2022**	1.2	3.67E-05	71,338	5.6	359,985
**2023**	1.5	4.73E-05	85,492	6.8	410,636
**2024**	1.8	6.01E-05	93,821	8.3	459,271
**2025**	2.1	9.38E-05	85,648	10.6	472,843
**Ca**					
**2021**	1.1	3.56E-05	71,717	5.3	349,276
**2022**	1.1	3.34E-05	64,900	5.7	363,933
**2023**	1.2	3.86E-05	70,455	6.2	373,664
**2024**	1.3	4.25E-05	67,747	6.7	371,531
**2025**	1.5	6.65E-05	63,312	7.5	335,511
**Mg**					
**2021**	0.4	1.10E-05	22,950	1.3	85,240
**2022**	0.4	1.21E-05	24,492	1.5	95,298
**2023**	0.5	1.66E-05	31,618	1.8	108,852
**2024**	0.6	1.96E-05	32,749	2.2	118,717
**2025**	0.6	2.84E-05	28,379	2.5	113,055
**B12**					
**2021**	0.1	1.90E-06	4011	0.2	13,371
**2022**	0.1	2.13E-06	4355	0.3	16,150
**2023**	0.1	2.32E-06	4475	0.3	18,633
**2024**	0.1	2.73E-06	4624	0.4	20,897
**2025**	0.1	3.94E-06	4007	0.5	21,236
**Underweight**					
**2021**	0.3	1.00E-05	1442	1.5	6615
**2022**	0.3	9.01E-06	1270	1.5	6890
**2023**	0.3	8.76E-06	1168	1.6	6959
**2024**	0.3	8.89E-06	1059	1.7	6827
**2025**	0.3	1.11E-05	819	1.9	6041
**Obese w/o excluding nutritional deficiencies**					
**2021**	3.7	1.36E-04	1,153,766	14.2	4,928,731
**2022**	4.0	1.45E-04	1,145,531	15.9	5,216,901
**2023**	4.3	1.61E-04	1,147,923	17.5	5,368,387
**2024**	4.6	1.81E-04	1,080,119	19.3	5,310,364
**2025**	5.0	2.70E-04	917,360	21.3	4,718,439
**Obese reference (excluding malnutrition)**					
**2021**	3.49	1.28E-4	1,073,192.00	12.9	4,397,287
**2022**	3.8	1.41E-4	1,055,752.00	14.8	4,547,295
**2023**	4.2	1.56E-4	1,057,964.00	16.2	4,663,042
**2024**	4.5	1.75E-4	988,133.00	17.9	4,575,700
**2025**	4.8	2.537E-4	833,214.00	19.5	4,014,224

*All patients with nutritional deficiencies at the first diagnosis of obesity with exception of underweight (>6 mo before first diagnosis of obesity).

Table S6. Representative laboratory values associated with metabolic diseases in cohorts of patients with obesity and nutritional deficiencies and the reference obese cohort without a history of nutritional deficiency. Data collected within three months of first diagnosis of obesity. Data represents Mean units of laboratory value, standard deviation (SD), and n=number of patients.

Variable	Mean/	Protein	Vit D	Ca	Mg	B12	Underw.	Obese ref.
**BMI**	kg/m²	36.73	36.39	36.43	36.6	36.01	29.47	33.78
	SD	7.96	7.29	7.47	7.21	7.12	7.37	6.61
	n	15,947	44,975	16,176	9898	9698	1065	35,813
**TG**	mg/mL	126.58	132.7	140.1	144.69	136.13	131.02	133.26
	SD	83.69	82.24	97.93	94.94	80.18	86.88	83.89
	n	2566	16,673	2943	1862	3459	218	8214
**HDL**	mg/mL	47.75	48.19	45.49	46.18	49.03	47.59	48.1
	SD	15.18	14.62	15.74	16.27	15.46	15.85	13.67
	n	2504	16,698	2859	1791	3464	214	8026
**HbA1c**	%	6.33	6.06	6.55	7.08	6.12	5.8	6.12
	SD	1.8	1.34	1.94	2.23	1.34	1.27	1.43
	n	3264	17,154	3744	2522	3566	242	9061
**F. Gluc.**	mg/dL	88.8	88.89	97.81	94.31	95.73	85.17	105.44
	SD	11.35	10.2	17.86	20.15	12.71	7.31	22.19
	n	138	96	81	41	17	10	1290
**Gluc.**	mg/dL	114.68	107.3	119.7	109.48	101.92	92	97.23
	SD	54.38	41.22	56.81	28.71	15.46	0	15.41
	n	9355	23,853	9755	137	25	10	62
**F. Insulin**	μUI/mL	12.5	20.24	20.0	43.9	19.0	0	34.57
	SD	12.02	10.73	1.41	0	5.42	0	16.8
	n	10	43	10	10	10	0	11
**Insulin**	μUI/mL	16.55	19.32	15.22	17.61	15.06	30.69	15.31
	SD	10.97	11.2	8.85	9.67	8.33	22.02	8.92
	n	54	665	64	50	72	10	688
**C-peptide**	ng/mL	2.61	2.98	1.85	2.35	2.1	14.5	3.24
	SD	1.26	1.62	1.25	1.58	0.7	1.0	1.78
	n	33	101	43	43	14	10	107
**ALT**	U/L	26.45	28.24	28.36	28.22	25.44	25.7	30.08
	SD	27.63	22.28	26.9	25.69	18.54	21.64	25.11
	n	8346	21,892	7936	5812	4729	412	13,340
**AST**	U/L	27.85	25.15	28.79	30.41	23.98	25.47	26.43
	SD	23.75	15.28	24.44	26.09	14.37	13.07	17.48
	n	8385	21,631	7849	5814	4727	401	12,601
**ALP**	U/L	96.53	83.87	87.42	86.55	77.59	127.2	91.14
	SD	50.74	42.52	42.18	42.9	26.78	85.98	57.59
	n	8353	20,833	7874	5778	4649	385	10,542
**Bilirubin**	mg/dL	0.52	0.53	0.56	0.61	0.55	0.49	0.56
	SD	0.61	0.31	0.55	0.63	0.37	0.34	0.39
	n	8025	19,999	7677	5704	4554	386	10,155
**SBP**	mmHg	126.93	125.2	128.4	131.3	127.3	114.0	124.3
	SD	18.55	17.52	19.6	20.31	16.22	15.05	19.55
	n	17,117	50,273	17,305	10,319	10,663	2248	45,632
**DBP**	mmHg	78.77	79.73	80.39	79.87	79.33	71.49	78.77
	SD	13.51	14.15	14.95	13.71	11.55	11.57	16.08
	n	17,120	50,270	17,304	10,318	10,661	2248	45,486
**Creat.**	mg/dL	0.9	0.82	0.81	0.84	0.85	0.72	0.83
	SD	0	0.31	0.4	0.39	0.25	0.23	0.29
	n	10	10	9415	6665	4972	368	15989
**eGFR**	mL/min/	105.61	98.02	95.42	72.17	93.05	108.25	96.21
	SD	25.72	21.35	17.54	16.68	17.59	26.51	22.23
	n	7355	18,499	145	18	67	194	11,154
**μAlb_Crea**	mg/g	36.52	30.63	44.03	47.27	36.78	12.37	40.64
	SD	68.55	54.57	75.63	80.8	68.64	12.62	77.01
	n	188	1113	262	177	229	23	563
**CRP**	mg/L	9.59	8.08	11.82	11.86	7.69	12.91	8.4
	SD	10.69	8.71	11.7	11.97	8.49	12.7	10.21
	n	360	1601	786	582	316	22	2393

Underw.: previous underweight status.

Table S7. Risk of diagnosis of metabolic syndrome (MetS) and its components in patients with obesity and nutritional deficiencies compared with patients with obesity but no history of nutritional deficiency.

Deficiency/ Component	Risk Deficient (%)	Risk Ref. (%)	RD	RR (95% CI)	OR (95% CI)	p value
**Protein**						
↑TG+↓HDL	8.16	5.93	0.02	1.38 (1.32–1.43)	1.41 (1.35–1.47)	5.8E-53
↑TG+AGR	13.10	8.99	0.04	1.46 (1.40–1.52)	1.53 (1.46–1.60)	3.4E-81
↑TG+HTN	25.69	17.98	0.08	1.43 (1.39–1.47)	1.58 (1.52–1.64)	2.1E-110
↓HDL+AGR	12.27	6.53	0.06	1.88 (1.80–1.95)	2.00 (1.91–2.09)	2.4E-206
↓HDL+HTN	20.87	15.88	0.05	1.31 (1.25–1.38)	1.40 (1.32–1.48)	6.0E-30
AGR+HTN	9.97	8.23	0.02	1.21 (1.18–1.24)	1.23 (1.20–1.27)	4.0E-56
MetS Diag.	0.11	0.14	-0.0003	0.77 (0.67–0.89)	0.77 (0.67–0.89)	4.1E-04
**Vit D**						
↑TG+↓HDL	12.42	8.43	0.04	1.47 (1.44–1.51)	1.54 (1.50–1.58)	3.6E-260
↑TG+AGR	15.32	8.78	0.07	1.75 (1.71–1.78)	1.88 (1.83–1.93)	<1E200
↑TG+HTN	16.80	16.02	0.01	1.05 (1.03–1.07)	1.06 (1.03–1.08)	4.1E-06
↓HDL+AGR	13.85	7.72	0.06	1.80 (1.75–1.84)	1.92 (1.87–1.98)	<1E200
↓HDL+HTN	11.68	12.43	-0.01	0.94 (0.91–0.97)	0.93 (0.90–0.96)	3.7E-05
AGR+HTN	15.97	8.83	0.07	1.81 (1.77–1.85)	1.96 (1.92–2.01)	<1E200
MetS Diag.	0.58	0.16	0.004	3.55 (3.28–3.85)	3.57 (3.29–3.87)	6.9E-236
**Ca**						
↑TG+↓HDL	8.39	6.96	0.01	1.21 (1.16–1.26)	1.22 (1.17–1.28)	2.1E-18
↑TG+AGR	12.72	8.88	0.04	1.43 (1.37–1.50)	1.50 (1.42–1.57)	9.1E-57
↑TG+HTN	25.50	17.38	0.08	1.47 (1.42–1.52)	1.63 (1.56–1.70)	1.3E-107
↓HDL+AGR	12.09	8.33	0.04	1.45 (1.37–1.53)	1.51 (1.43–1.61)	4.7E-41
↓HDL+HTN	19.99	15.92	0.04	1.26 (1.20–1.31)	1.32 (1.25–1.39)	6.3E-23
AGR+HTN	10.50	8.43	0.02	1.25 (1.21–1.28)	1.27 (1.24–1.31)	1.6E-59
MetS Diag.	0.10	0.12	-0.0002	0.82 (0.71–0.95)	0.82 (0.71–0.95)	0.006
**Mg**						
↑TG+↓HDL	9.69	6.67	0.03	1.45 (1.37–1.54)	1.50 (1.41–1.60)	1.2E-37
↑TG+AGR	13.79	8.38	0.05	1.65 (1.54–1.75)	1.75 (1.63–1.88)	3.4E-52
↑TG+HTN	28.13	18.14	0.10	1.55 (1.48–1.63)	1.77 (1.65–1.89)	3.0E-65
↓HDL+AGR	12.12	8.66	0.03	1.40 (1.27–1.54)	1.46 (1.31–1.62)	4.4E-12
↓HDL+HTN	21.26	16.90	0.04	1.26 (1.17–1.35)	1.33 (1.21–1.45)	4.7E-10
AGR+HTN	11.63	8.40	0.03	1.38 (1.33–1.44)	1.44 (1.37–1.50)	2.5E-54
MetS Diag.	0.08	0.12	0.0004	0.64 (0.49–0.83)	0.64 (0.49–0.83)	0.0007
**B12**						
↑TG+↓HDL	10.16	7.39	0.03	1.37 (1.29–1.47)	1.42 (1.32–1.52)	9.6E-22
↑TG+AGR	15.29	9.08	0.06	1.68 (1.59–1.79)	1.81 (1.69–1.93)	2.6E-67
↑TG+HTN	16.51	17.43	-0.01	0.95 (0.89–1.00)	0.94 (0.87–1.01)	0.07
↓HDL+AGR	15.11	8.23	0.07	1.84 (1.69–1.99)	1.98 (1.81–2.18)	1.4E-48
↓HDL+HTN	13.91	15.63	-0.02	0.89 (0.82–0.96)	0.87 (0.79–0.96)	0.003
AGR+HTN	15.74	9.33	0.06	1.69 (1.60–1.77)	1.81 (1.71–1.92)	5.0E-91
MetS Diag.	0.43	0.15	0.002	2.82 (2.23–3.57)	2.83 (2.24–3.58)	1.4E-19
**Underw.**						
↑TG+↓HDL	9.55	7.91	0.02	1.21 (0.97–1.50)	1.23 (0.97–1.56)	0.088
↑TG+AGR	14.06	6.37	0.08	2.21 (1.78–2.74)	2.40 (1.90–3.05)	1.1E-13
↑TG+HTN	15.35	10.87	0.04	1.41 (1.17–1.71)	1.49 (1.19–1.85)	0.00037
↓HDL+AGR	11.67	11.25	0.004	1.04 (0.80–1.35)	1.04 (0.77–1.40)	0.78
↓HDL+HTN	11.67	11.25	0.004	1.04 (0.80–1.35)	1.04 (0.77–1.40)	0.78
AGR+HTN	13.32	5.41	0.08	2.46 (2.01–3.02)	2.69 (2.16–3.35)	8.6-20
MetS Diag.	0.56	0.18	0.003	3.05 (2.26–4.12)	3.06 (2.27–4.13)	1.3E-14

Metabolic Syndrome components: diagnosis of obesity plus: 1: hypertriglyceridemia and low HDL (↑TG+↓HDL); 2: hypertriglyceridemia and abnormal glucose regulation (↑TG+ AGR); 3: hypertriglyceridemia and hypertension (↑TG+HTN); 4: low HDL and abnormal glucose regulation (↓HDL+AGR); 5: low HDL and hypertension (↓HDL+HTN); 6: abnormal glucose and hypertension (AGR+HTN). Diagnosis of metabolic syndrome (MetS Diag.). RD: Risk difference; RR: Risk ratio; OR: Odds ratio. In large cohorts, risk differences (RD) <1% (0.01) may have limited clinical relevance despite statistical significance. Underw: history of underweight at least 6 months before the first diagnosis of obesity. Data based on the initial PSM model based on obesity status/severity.

Table S8. Sensitivity analyses show the risk ratio (RR) and 95% confidence interval (95% IC) of metabolic outcomes among patients with obesity and nutritional deficiencies relative to a reference cohort of patients with obesity without nutritional deficiencies. Propensity score matching models (PSM) using body mass index (BMI) and BMI plus laboratory values (BMI + labs) were evaluated against the initial PSM model based on obesity status/severity to assess the robustness of the findings. RR, risk ratio.

		**PSM Model**			
**Condition**	Deficiency	Initial RR (95% CI)	BMI RR (95% CI)	BMI + Labs RR (95% CI)	*p*
**Obesity Class 2**	Protein	0.93 (0.89-0.97)	1.40 (1.34-1.45)	1.39 (1.34-1.45)	***
	Vit. D	3.35 (3.25-3.46)	5.13 (4.98-5.28)	5.12 (4.98-5.28)	***
	Ca	1.14 (1.09-1.20)	1.82 (1.74-1.91)	1.74 (1.66-1.82)	***
	Mg	1.09 (1.01-1.18)	1.77 (1.63-1.91)	1.87 (1.73-2.03)	***
	B12	2.91 (2.68-3.15)	5.19 (4.76-5.65)	5.75 (5.26-6.30)	***
	Underw.	2.95 (2.61-3.33)	2.13 (1.69-2.68)	N/A	***
**Obesity Class 3**	Protein	1.51 (1.45-1.57)	2.78 (2.67-2.89)	2.90 (2.78-3.02)	***
	Vit. D	3.21 (3.11-3.32)	6.05 (5.86-6.24)	5.95 (5.76-6.14)	***
	Ca	1.64 (1.56-1.72)	3.02 (2.87-3.18)	3.11 (2.95-3.27)	***
	Mg	1.69 (1.56-1.83)	2.92 (2.67-3.18)	3.34 (3.05-3.66)	***
	B12	4.02 (3.64-4.43)	6.91 (6.21-7.69)	7.86 (7.02-8.81)	***
	Underw.	2.70 (2.37-3.07)	1.31 (1.02-1.68)	N/A	***; 0.035
**Prediabetes**	Protein	1.04 (1.02-1.06)	1.12 (1.10-1.14)	1.10 (1.08-1.12)	***
	Vit. D	2.41 (2.38-2.44)	2.59 (2.55-2.62)	2.65 (2.62-2.68)	***
	Ca	1.07 (1.04-1.09)	1.17 (1.14-1.19)	1.09 (1.06-1.11)	***
	Mg	0.94 (0.91-0.98)	1.00 (0.97-1.04)	1.06 (1.03-1.10)	0.001-0.947
	B12	2.26 (2.17-2.35)	2.32 (2.23-2.40)	2.28 (2.20-2.36)	***
	Underw.	1.33 (1.25-1.42)	1.01 (0.90-1.14)	N/A	***; NS
**Diabetes**	Protein	1.11 (1.08-1.13)	1.39 (1.36-1.42)	1.45 (1.42-1.49)	***
	Vit. D	1.22 (1.20-1.24)	1.63 (1.60-1.66)	1.68 (1.65-1.72)	***
	Ca	1.09 (1.06-1.12)	1.43 (1.39-1.46)	1.43 (1.39-1.46)	***
	Mg	1.27 (1.22-1.32)	1.51 (1.45-1.58)	1.58 (1.51-1.65)	***
	B12	0.97 (0.92-1.03)	1.37 (1.30-1.44)	1.38 (1.31-1.45)	NS; ***
	Underw.	1.05 (0.94-1.17)	1.48 (1.18-1.86)	N/A	NS; ***
**MetS Diag.**	Protein	0.77 (0.67-0.89)	1.30 (1.14-1.49)	1.10 (0.98-1.24)	0.0004; 0.121
	Vit. D	3.55 (3.28-3.85)	6.22 (5.76-6.72)	6.55 (6.06-7.09)	***
	Ca	0.82 (0.70-0.95)	1.49 (1.29-1.72)	1.30 (1.14-1.49)	0.007; <0.001
	Mg	0.64 (0.49-0.83)	1.46 (1.14-1.86)	1.46 (1.14-1.86)	0.0007; 0.0028
	B12	2.82 (2.23-3.57)	5.95 (4.63-7.65)	5.33 (4.20-6.76)	***
	Underw.	3.05 (2.26-4.12)	0.84 (0.43-1.64)	N/A	***; 0.611
**MASLD**	Protein	1.47 (1.12-1.92)	2.06 (1.69-2.50)	1.92 (1.58-2.32)	***
	Vit. D	2.12 (2.05-2.19)	2.50 (2.44-2.56)	2.43 (2.37-2.49)	***
	Ca	1.34 (1.28-1.40)	1.54 (1.48-1.60)	1.40 (1.35-1.45)	***
	Mg	1.42 (1.32-1.52)	1.75 (1.64-1.86)	1.68 (1.58-1.78)	***
	B12	1.90 (1.76-2.06)	2.13 (1.99-2.29)	2.06 (1.92-2.21)	***
	Underw.	1.79 (1.42-2.26)	1.43 (1.20-1.72)	N/A	***
**MASH**	Protein	1.21 (0.66-2.22)	2.84 (1.63-4.93)	2.29 (1.37-3.83)	NS; ***
	Vit. D	2.42 (2.23-2.63)	3.20 (2.98-3.43)	3.27 (3.05-3.51)	***
	Ca	1.09 (0.96-1.24)	1.53 (1.37-1.72)	1.40 (1.25-1.56)	0.016; ***
	Mg	1.00 (0.82-1.23)	1.43 (1.18-1.74)	1.45 (1.20-1.75)	0.066; ***
	B12	2.09 (1.66-2.63)	2.63 (2.12-3.26)	2.38 (1.94-2.92)	***
	Underw.	1.44 (0.79-2.63)	1.41 (0.86-2.30)	N/A	NS

***P<0.001; NS: non-significant. First significance value corresponds to the initial PMS model (obesity/obesity severity) and second significance value corresponds to the PSM for BMI and BMI + Labs. MetS Diag.: metabolic syndrome diagnosis.

Table S9. Sensitivity analyses show the hazard ratio (HR) and 95% confidence interval (95% CI) of metabolic outcomes among patients with obesity and nutritional deficiencies relative to a reference cohort of patients with obesity without nutritional deficiencies. Propensity score matching models (PSM) using body mass index (BMI) and BMI plus laboratory values (BMI + labs) were evaluated against the initial PSM model based on obesity status/severity to assess the robustness of the findings.

		**PSM Model**			
**Deficiency**	Outcome	Initial HR (95% CI)	BMI HR (95% CI)	BMI + Labs HR	Significance
**Protein**	Obesity class 1	1.13 (1.08-1.18)	1.74 (1.67-1.81)	1.69 (1.63-1.76)	***
	Obesity class 2	1.83 (1.76-1.90)	3.46 (3.33-3.61)	3.55 (3.41-3.69)	***
	Prediabetes	1.20 (1.18-1.23)	1.35 (1.33-1.38)	1.72 (1.68-1.76)	***
	Diabetes	1.26 (1.23-1.29)	1.67 (1.63-1.71)	1.31 (1.29-1.34)	***
	MetS Diag.	0.91 (0.79-1.05)	3.12 (1.79-5.44)	1.33 (1.18-1.51)	NS; ***
	MASLD	1.55 (1.18-2.04)	1.60 (1.41-1.82)	2.08 (1.72-2.52)	***
	MASH	1.30 (0.70-2.38)	2.26 (1.86-2.76)	2.46 (1.47-4.11)	NS; ***
**Vit. D**	Obesity class 1	3.16 (3.06-3.26)	4.94 (4.80-5.08)	4.87 (4.72-5.01)	***
	Obesity class 2	3.04 (2.94-3.14)	5.84 (5.65-6.02)	5.66 (5.48-5.85)	***
	Prediabetes	2.30 (2.27-2.33)	2.58 (2.55-2.61)	1.63 (1.60-1.67)	***
	Diabetes	1.13 (1.11-1.15)	1.60 (1.57-1.63)	2.62 (2.58-2.65)	***
	MetS Diag.	3.29 (3.03-3.57)	3.03 (2.83-3.25)	6.17 (5.70-6.68)	***
	MASLD	2.02 (1.96-2.09)	5.89 (5.45-6.36)	3.09 (2.88-3.31)	***
	MASH	2.28 (2.10-2.48)	2.40 (2.34-2.46)	2.32 (2.27-2.38)	***
**Ca**	Obesity class 1	1.23 (1.17-1.29)	1.98 (1.89-2.07)	1.89 (1.80-1.98)	***
	Obesity class 2	1.77 (1.69-1.86)	3.29 (3.13-3.46)	3.39 (3.22-3.57)	***
	Prediabetes	1.14 (1.11-1.16)	1.27 (1.24-1.30)	1.54 (1.50-1.59)	***
	Diabetes	1.15 (1.12-1.18)	1.54 (1.50-1.58)	1.18 (1.16-1.21)	***
	MetS Diag.	0.86 (0.74-1.00)	1.62 (1.41-1.87)	1.41 (1.23-1.61)	p=0.049; ***
	MASLD	1.44 (1.38-1.51)	1.65 (1.47-1.85)	1.51 (1.35-1.69)	***
	MASH	1.17 (1.03-1.33)	1.66 (1.60-1.72)	1.51 (1.46-1.57)	***
**M**g	Obesity class 1	1.45 (1.34-1.57)	2.36 (2.18-2.56)	2.41 (2.22-2.62)	***
	Obesity class 2	2.23 (2.05-2.42)	3.88 (3.56-4.24)	4.31 (3.93-4.72)	***
	Prediabetes	1.20 (1.16-1.24)	1.31 (1.26-1.35)	1.99 (1.90-2.07)	***
	Diabetes	1.60 (1.53-1.66)	1.96 (1.88-2.05)	1.34 (1.29-1.39)	***
	MetS Diag.	0.81 (0.63-1.06)	1.94 (1.51-2.48)	1.90 (1.48-2.43)	NS; ***
	MASLD	1.71 (1.59-1.84)	1.76 (1.45-2.15)	2.10 (1.97-2.24)	***
	MASH	1.21 (0.99-1.48)	2.10 (1.97-2.25)	1.84 (1.52-2.23)	NS; ***
B12	Obesity class 1	3.06 (2.82-3.32)	5.49 (5.03-5.99)	6.08 (5.55-6.66)	***
	Obesity class 2	4.24 (3.84-4.69)	7.30 (6.55-8.13)	8.33 (7.43-9.34)	***
	Prediabetes	2.38 (2.28-2.48)	2.49 (2.40-2.59)	1.46 (1.39-1.55)	***
	Diabetes	0.99 (0.94-1.05)	1.46 (1.38-1.54)	2.42 (2.33-2.51)	NS; ***
	MetS Diag.	2.91 (2.30-3.69)	6.08 (4.72-7.82)	5.57 (4.38-7.07)	***
	MASLD	1.97 (1.82-2.13)	2.74 (2.21-3.39)	2.16 (2.01-2.32)	***
	MASH	2.12 (1.68-2.67)	2.24 (2.08-2.40)	2.49 (2.03-3.06)	***
**Underw.**	Obesity class 1	4.27 (3.78-4.83)	3.36 (2.65-4.24)	N/A	***
	Obesity class 2	3.82 (3.35-4.35)	2.09 (1.62-2.69)	N/A	***
	Prediabetes	1.76 (1.65-1.88)	1.45 (1.28-1.64)	N/A	***
	Diabetes	1.37 (1.23-1.53)	2.27 (1.80-2.86)	N/A	***
	MetS Diag.	3.98 (2.94-5.40)	1.24 (0.63-2.43)	N/A	***; NS
	MASLD	2.23 (1.76-2.83)	1.83 (1.12-3.01)	N/A	***
	MASH	1.91 (1.04-3.51)	1.75 (1.46-2.10)	N/A	***

***P<0.001; NS: non-significant. First significance value corresponds to the initial PMS model (obesity/obesity severity) and second significance value corresponds to the PSM for BMI and BMI + Labs. MetS Diag.: metabolic syndrome diagnosis.
